# QTL mapping for seed density per silique in *Brassica napus*

**DOI:** 10.1038/s41598-023-28066-5

**Published:** 2023-01-14

**Authors:** Jifeng Zhu, Lei Lei, Weirong Wang, Jianxia Jiang, Xirong Zhou

**Affiliations:** Key Laboratory of Germplasm Innovation and Genetic Improvement of Grain and Oil Crops (Co-Construction By Ministry and Province), Ministry of Agriculture and Rural Affairs, Crop Breeding and Cultivation Research Institute, Shanghai Academy of Agricultural Sciences, Shanghai, 201403 China

**Keywords:** Genetic linkage study, Quantitative trait, Sequencing

## Abstract

Seed density per silique (SDPS) and valid silique length (VSL) are two important yield-influencing traits in rapeseed. SDPS has a direct or indirect effect on rapeseed yield through its effect on seed per silique. In this study, a quantitative trait locus (QTL) for SDPS was detected on chromosome A09 using the QTL-seq approach and confirmed via linkage analysis in the mapping population obtained from 4263 × 3001 cross. Furthermore, one major QTL for SDPS (*qSD.A9-1*) was mapped to a 401.8 kb genomic interval between SSR markers *Nys9A190* and *Nys9A531*. In the same genomic region, a QTL (*qSL.A9)* linked to VSL was also detected. The phenotypic variation of *qSD.A9-1* and *qSL.A9* was 53.1% and 47.6%, respectively. Results of the additive and dominant effects demonstrated that the expression of genes controlling SDPS and VSL were derived from a different parent in this population. Subsequently, we identified 56 genes that included 45 specific genes with exonic (splicing) variants. Further analysis identified specific genes containing mutations that may be related to seed density as well as silique length. These genes could be used for further studies to understand the details of these traits of rapeseed.

## Introduction

Rapeseed (*Brassica napus* L.) is a major oilseed crop and is also used as animal feed and in biodiesel production globally. Developing a high-yielding rapeseed variety has been an important goal to meet the increasing vegetable oil demand^1,2^. Rapeseed yield should be improved by increasing the seed number per silique (SPS), which is one of the three direct yield-related components, with the other being silique number and seed weight^3^. SPS is influenced by valid silique length (VSL) and seed density per silique (SDPS)^4^. VSL or SDPS has a significant positive relationship with SPS and affects rapeseed yield directly or indirectly through effects on SPS.

The silique traits, including SPS, SDPS, and VSL, are complex traits and controlled by quantitative trait loci (QTL) with high heritability^4,5^. Application of molecular markers and DNA sequencing are powerful tools to analyze genomes and map genes related to agronomic traits^4,6^. In recent years, QTL associated with silique traits have been identified by using different QTL mapping approaches in different populations of *B. *napus^3–5,7,8^. Hussain et al. (2022)^8^ detected an association of 13 loci with VSL in a genome-wide association study conducted using 331 core accessions planted in 10 environments. Wang et al. (2016)^4^ obtained 60 consensus QTL for six silique-related traits, including 11 for VSL, 6 for SPS, and 5 for SDPS, by consensus map construction and QTL comparison. Since *B. napus* has a complex genome, so far only a few QTL associated with SPS or VSL have been mapped or cloned and functionally characterized^2,3,8–11^. *BnaA.ARF18*, the first QTL that affects both seed weight and VSL was identified on chromosome A9 of rapeseed by fine mapping and association analysis^11^. In another study, *BnaC9.SMG7b* associated with SPS was identified on chromosome C9 of rapeseed using a map-based cloning strategy^10^. Furthermore, two other major QTL associated with VSL were identified on chromosome C7 (*BnaC7.ROT3*) and A9 (*BnaA9.CYP78A9*) of rapeseed and were cloned and characterized using map-based cloning^2,9^. Although a few candidate genes for silique traits have been cloned and functionally characterized, key candidate genes for silique traits, especially for SDPS, remain undiscovered. Thus, it is important to identify novel QTL related to SDPS and its related traits for fine mapping and cloning to accelerate the breeding of high-yield varieties of rapeseed.

Next-generation sequencing (NGS) is a useful tool to study the genetic architecture of complex traits in plants at low cost. QTL-seq is a method that combines bulked segregant analysis (BSA) and NGS to effectively and rapidly identify QTL for different traits^6^. The present study employed F_2:3_ populations derived from two inbred lines (3001 and 4263) with significant differences in silique traits, particularly SDPS, to detect the QTL for SDPS and its related traits by combining QTL-seq and linkage mapping. This study mainly aimed to (1) identify the genomic region of QTL for SDPS, (2) construct a linkage map of the candidate region with simple sequence repeat (SSR) markers, (3) validate and fine map major QTL using the linkage map by linkage association analysis, and (4) identify the candidate genes related to SDPS and its related traits by conditional QTL analysis.

## Results

### Phenotypic analysis and bulk construction

**Figure 1:**
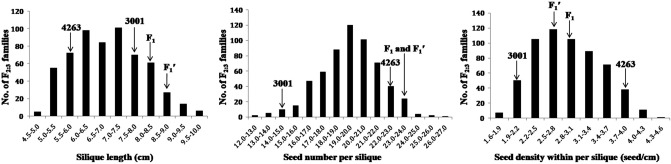
Distribution of the SDPS and its related traits phenotype in F_2:3_ population.

Phenotypic performance and frequency distribution of SDPS and its related traits in all F_1_ plants, F_2_ population, F_3_ families, and two parental lines were analyzed (Fig. 1 and Fig.S1). Two parental lines (3001 and 4263) had significantly different phenotypes of SDPS and its related traits; 4263 showed a higher SDPS and more seeds than 3001 in different years, whereas 3001 lines had longer siliques than 4263 lines. The SDPS of F_1_ (obtained from 3001 × 4263 cross [2.85 seeds/cm]) and F_1_′ (obtained from 4263 × 3001 cross [2.72 seeds/cm]) plants was lower than 4263 but higher than 3001, whereas the SPS and VSL of both plants obtained after the reciprocal crosses were significantly higher than those of two parental lines. Both F_2_ populations and F_2:3_ families demonstrated pronounced phenotypic variations and transgressive segregation of SDPS and its related traits. In the F_2:3_ families, the broad-sense heritability estimate of SPDS and VSL were 0.907 and 0.862, respectively, in the parent–offspring regression analysis, indicating that SPDS and VSL were stably inherited. In contrast, SPS demonstrated a broad-sense heritability estimate of 0.293, suggesting sensitivity to environmental variation.

Phenotypic data of SDPS, SPS, and VSL showed continuous distributions, and their skewness and kurtosis values were < 1.0 in the segregation populations (Table 1), indicating the quantitative inheritance of these traits. The maximum and minimum SDPS values of F_2_ plants were 4.90 seeds/cm and 1.23 seeds/cm, respectively, whereas, at the same time, the SDPS values of 4263 and 3001 parental lines were 4.14 seeds/cm and 2.24 seeds/cm, respectively (Table 1 and Fig.S1). F_2:3_ families also demonstrated a wide distributed SDPS values that ranged from less than that of 3001 to higher than that of 4263. These SDPS data indicated the suitability of subsequent QTL-seq analysis. To construct the high-seed density trait (HT) and low-seed density trait (LT) bulks, F_2_ individuals (40 each of HT and LT) were selected. Further, the SDPS of 40 plants in the HT and LT bulks was > 4.01 seeds/cm and < 2.53 seeds/cm, respectively.

**Table 1:** Statistical analysis of seed density within per silique and its related traits in F_2_ population and F_2:3_ family.

Sample	Size^a^	Trait	Mean	Range^b^	Variance	Skewness	Kurtosis	W-test^c^	*p*-value^d^
F_2_	780	SPS^e^	20.84 ± 3.36	8.58–30.36	11.27	− 0.57	0.38	0.969	0.0000
		VSL^f^	7.12 ± 1.19	4.15–10.60	1.41	0.07	− 0.57	0.976	0.0001
		SDPS^g^	2.98 ± 0.61	1.23–4.90	0.37	0.65	− 0.16	0.940	0.0000
F_2:3_	573	SPS^e^	19.42 ± 2.21	12.16–26.48	4.88	− 0.18	0.13	0.987	0.6981
		VSL^f^	6.92 ± 1.07	4.67–9.98	1.15	0.32	− 0.49	0.966	0.0000
		SDPS^g^	2.89 ± 0.54	1.71–4.39	0.29	0.26	− 0.76	0.961	0.0000

^a^Size of the sample population. ^b^Range of the minimum value and maximum value for the trait. ^c^W-test is the Shapiro Wilk W-statistic for the test of normality. ^d^*p*-value is the *p*-value for W-test of normality. ^e^SPS is seed number per silique. ^f^VSL is valid silique length (cm). ^g^SDPS is seed density within per silique (seed/cm).

### Resequencing and mapping of reads

For Illumina sequencing, libraries were prepared using HT and LT bulks and the two parental lines. A total of 216,731,002, 223,784,954, 220,390,200, and 209,578,748 clean reads were obtained for HT bulk, LT bulk, high-SDPS parent (4263), and low-SDPS parent (3001), respectively. When these reads were aligned, the read depth and coverage observed were 24.81 × and 99.65%, 25.36 × and 99.66%, 25.32 × and 96.79%, and 24.12 × and 96.16% for HT bulk, LT bulk, 4263, and 3001, respectively (Table 2), based on the Ningyou7 rapeseed genome sequence^12^. In comparison to the reference genome, HT bulk and LT bulk were identified with a total of 5,301,640 and 5,313,578 genome-wide single nucleotide polymorphisms (SNPs) and 1,159,752 and 1,163,307 insertions or deletions (InDels), respectively (Table S1 and Fig. S2).

**Table 2:** Summary of the sequencing results for two bulks and their parental lines.

Samples	Clean reads	Clean base (Gb)	Read alignment (%)	Average depth ( ×)
LT bulk	223,784,954	33.31	99.66	25.36
HT bulk	216,731,002	32.27	99.65	24.81
3001	209,578,748	31.21	96.16	24.12
4263	220,390,200	32.82	96.79	25.32

### Identification of SDPS-related genomic region using QTL-seq

**Figure 2:**
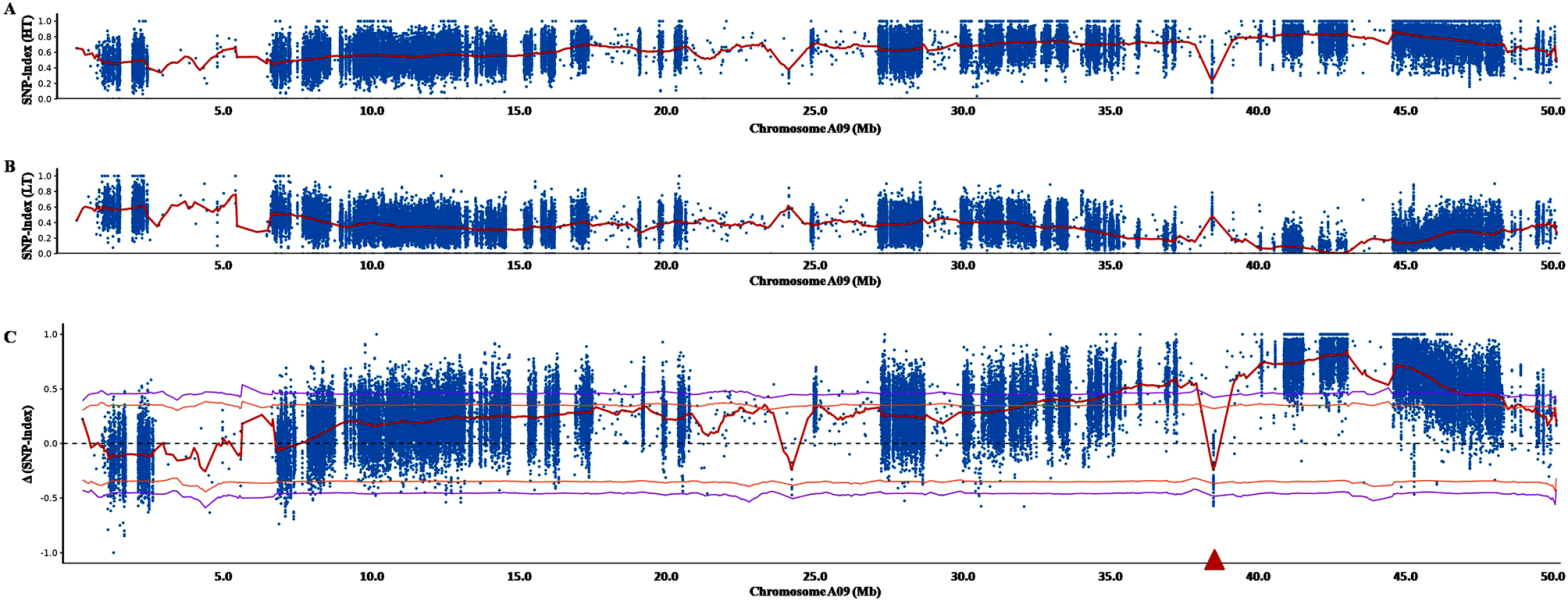
QTL identification for SDPS in F_2_ lines by QTL-seq. (**A**–**C**) represent the SNP-index plots for HT bulk and LT bulk, and the Δ(SNP-index) plot of rapeseed chromosome A09 from QTL-seq analysis, respectively. Δ(SNP-index) plot with statistical confidence interval under the null hypothesis of no QTL (orange, *P* < 0.01; purple, *P* < 0.05).The X-axis and Y-axis represent the rapeseed chromosomes and the SNP-index, respectively.

To identify the genomic region associated with SDPS, the Δ(SNP-index) was calculated and plotted against the genome positions based on the information of the SNP index for each bulk (Fig. 2). The Δ(SNP-index) was calculated by subtracting the SNP-index of the LT bulk from that of the HT bulk and plotted across all rapeseed chromosomes to map the putative genomic region related to SDPS. The region located between 31.71 Mb and 49.20 Mb on chromosome A09 was detected at 95% significance (Fig. 2C), and one SDPS-associated QTL was identified at the 17.49 Mb region of chromosome A09.

### Marker development and QTL fine mapping

**Figure 3:**
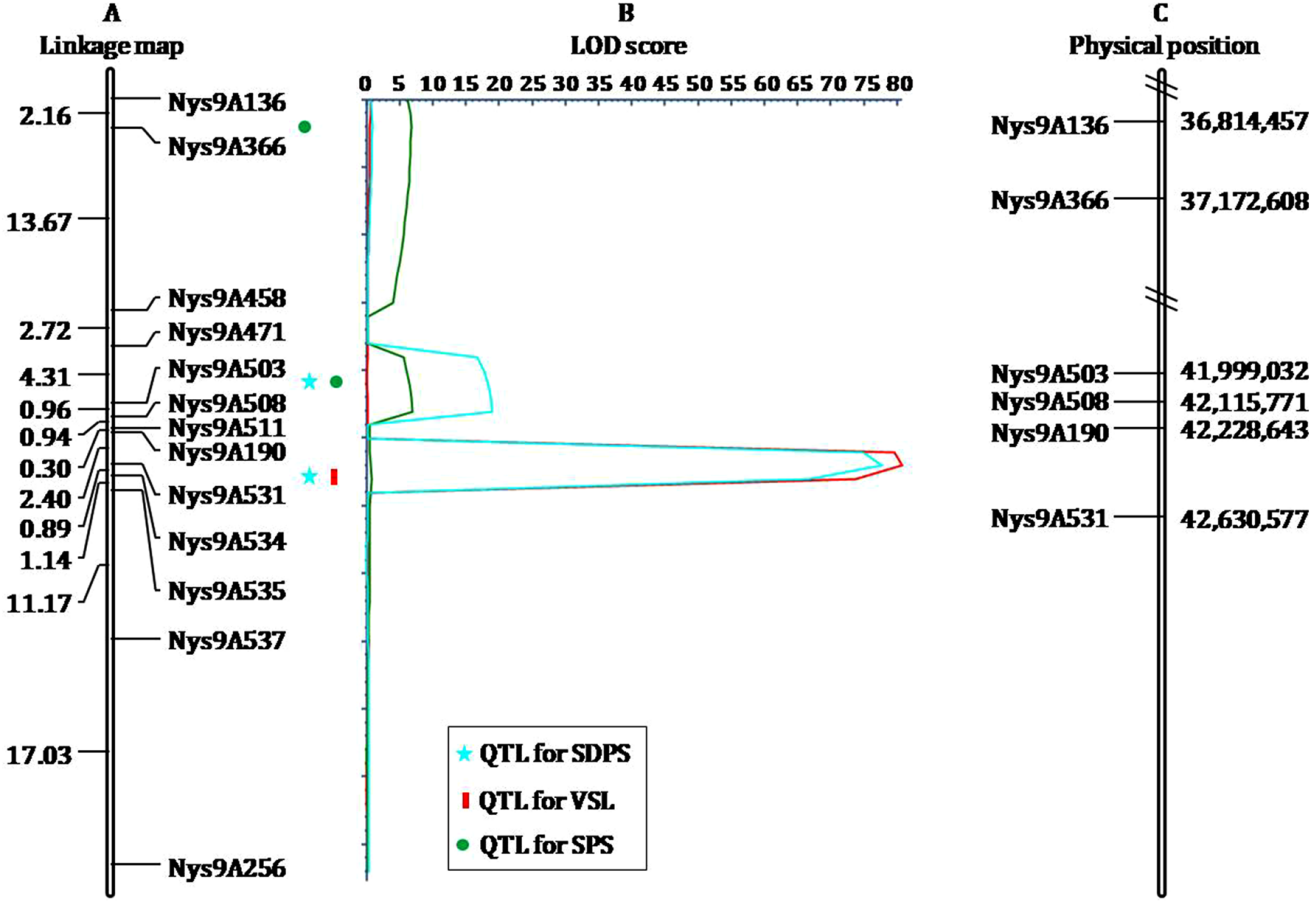
QTL fine mapping of SDPS and its related traits in F_2:3_ families from 4263 × 3001 cross. (**A**) is the chromosome A09 with map interval sizes in Kosambi centiMorgan (cM) units at left, and markers at right. (**B**) is the logarithm of odds (LOD) score of the detected QTL for SDPS and its related traits in F_2:3_ families on chromosome A09. (**C**) is the physical position of the markers linked with the detected QTL for SDPS and its related traits on chromosome A09 of the Ningyou7 reference genome.

To fine-map the candidate region, 383 SSR markers were developed and genotyped in two parental lines. From these markers, 15 polymorphic markers (Table 3) with clear and steady bands were selected to analyze 847 F_2_ individuals to construct genetic linkage maps. This led to the identification of a QTL for SDPS, named *qSD.A9-1*, that accounted for 53.1% of the phenotypic variations (PVs) in the F_2:3_ families. *qSD.A9-1* was mapped in a 2.40 cM genetic interval between *Nys9A190* and *Nys9A531* on the linkage map and a 401.8 kb physical interval between 42.22 Mb and 42.63 Mb on rapeseed chromosome A09 of the Ningyou7 reference genome^12^. Coincidentally, another QTL, named *qSL.A9,* was identified for VSL in the same region as *qSD.A9-1*, explaining a PV of 47.6% in this population. Furthermore, *qSD.A9-1* had additive and dominant effects of 0.4 and -0.2, respectively, whereas *qSL.A9* had additive and dominant effects of -1.0 and 0.3, respectively, indicating that the major QTL in the region between *Nys9A190* and *Nys9A531* controlled the expression of SDPS from 4263 and VSL from 3001. We also identified 56 genes in this region predicted based on the annotations of Ningyou7 reference genome^12^ (Table S2). Additionally, we detected the following minor QTL: (1) *qSD.A9-2* (near *qSD.A9-1*) for SDPS, with a PV of 10.0% mapped in a 0.96 cM interval between *Nys9A503* and *Nys9A508*, (2) *qSP.A9-2* for SPS, which was mapped in the same region as *qSD.A9-2* and showed a PV of 7.7%, and (3) *qSP.A9-1* for SPS that was located in a 2.16 cM interval between *Nys9A136* and *Nys9A366* on the same chromosome and accounted for 7.6% of PV (Fig. 3 and Table 4).

**Table 3:** Polymorphic markers used for linkage analysis.

Markers	Primer sequences		Location^a^
	Forward (5′-3′)	Reverse (5′-3′)	
Nys9A136	TCTCCAACTCGATTCCTTTA	CCCACAAACACATGTAATCA	36814457–36814567
Nys9A366	GTGGAGAAAAAGGAAGGATT	TGATACCCAAAAATGGTAGC	37172608–37172896
Nys9A458	GCATTGGTCTTCAAAGAAAC	GCTGGCATTTAGTGATTTTC	40879333–40879599
Nys9A471	ATCTATCATCTCCATGTGCC	GTACCACCACCACATAGCTT	41227405–41227580
Nys9A503	GAACAAAAGGATTCACAAGG	ACTCGACAGAAAGGACAAGA	41999032–41999244
Nys9A508	GTTGCTTGATTTAGGGTTTG	CGAAAGTAAAGCTCTCGTGT	42115771–42116068
Nys9A511	ATGGAACTCAGAGATTGGTG	AGCAGAGTCTGACGAAACAT	42196513–42196746
Nys9A190	TTTTAGAACGGAGCGTAAAG	TAGGATTTGGTGGAGAAGAA	42228643–42228762
Nys9A531	GTTCTCATCTTTCTTGACGC	TGGTGCTTTAGGTTTCACTT	42630577–42630786
Nys9A534	AGACCCAAGAGGAAAAGAAC	ACCTTCCATTGTACACGAAC	42683900–42684063
Nys9A535	CTTTCTCGATCCTTCATACG	CCGACCACTGAAAAATTAAC	42689011–42689169
Nys9A537	CACAGGAACGGTTAGTTCTC	CGGAGAAGTAAGTCTCCAAA	42719951–42720183
Nys9A558	CTACTACACCTGCTGCACAA	AACACGAAGCTCTTTTCTTG	43130463–43130633
Nys9A256	CAGTGGCCTCTAGTGTTTTC	AAGTATAACCGCCATACCCT	44757606–44757796
Nys9A258	TGGGAATAGGATACCTTGTG	AAAGTCAGCGAAGATGTGTT	44795100–44795254

^a^Location is the marker position on chromosome A09 of the reference genome sequence.

**Table 4:** Statistics of seed density within per silique and its related traits QTL identified on chromosome A09 in F_2:3_ families.

QTL	Marker Interval	Distance (cM)^a^	LOD	PV (%)^b^	Add^c^	Dom^d^
*qSD.A9-1*	Nys9A190-Nys9A531	2.40	77.6	53.1	0.4	− 0.2
*qSD.A9-2*	Nys9A503-Nys9A508	0.96	18.9	10.0	0.2	0.0
*qSP.A9-1*	Nys9A136-Nys9A366	2.16	6.6	7.6	0.7	0.0
*qSP.A9-2*	Nys9A503-Nys9A508	0.96	6.7	7.7	0.7	− 0.2
*qSL.A9*	Nys9A190-Nys9A531	2.40	80.7	47.6	− 1.0	0.3

^a^The distance between the left and right marker of the mapping QTL in F_2:3_ families. ^b^PV means the phenotypic variation of each QTL for SDPS or its related trait. ^c^Add is the additive effect. ^d^Dom is the dominant effect.

### Sequence analysis of the major QTL for SDPS and VSL

When we screened 822 SNPs and 234 InDels in the 56 predicted genes (Table S2), 45 predicted genes in the region of *qSD.A9-1* and *qSL.A9* were exonic or splicing variants. Among these, 36 predicted genes were exonic variants with amino acid changes, including 33, five, two, and one with nonsynonymous single nucleotide variation, non-frameshift insertion or deletion, frameshift insertion/deletion, and splicing variant, respectively (Table S2). These variations may be related to altered seed density and silique length in rapeseed. Using the Ningyou7 annotation, the following 11 predicted genes were found to encode proteins associated with plant growth and development: (1) *ChrA09g005039* encoding a pentatricopeptide (PPR) repeat, (2) *ChrA09g005043* encoding an skp1 family protein, (3) *ChrA09g005048*, (4) *ChrA09g005056*, and (5) *ChrA09g005072* encoding ring finger proteins, (6) *ChrA09g005049* encoding a 2OG-Fe(II) oxygenase superfamily protein, (7) *ChrA09g005059* encoding an F-box-like protein, (8) *ChrA09g005075* encoding an auxin response factor, (9) *ChrA09g005077* encoding an RNA recognition motif, (10) *ChrA09g005091* encoding a protein kinase, and (11) *ChrA09g005092* encoding a B3 DNA binding protein (Table S3). Except for *ChrA09g005043*, the coding sequence of all other genes showed variations between the two parents (Table 5).

**Table 5:** Sequences alignment for CDS and amino acid of the part genes related to *qSD.A9-1* (*qSL.A9*) between 4263 and 3001.

GeneID	The base for CDS			Amino acid change		
	Mutation position	4263	3001	Mutation position	4263	3001
chrA09g005039	346	G	A	116	V	M
	523	A	G	175	T	A
	1003	C	G	335	P	A
	1087	C	T	363	H	Y
chrA09g005048	73	C	T	25	L	F
chrA09g005049	122	A	C	41	K	T
	370	A	T	124	T	S
	763	G	A	255	G	R
	1014	G	C	338	K	N
chrA09g005056	25	T	C	9	F	L
chrA09g005059	315	C	A	105	D	E
	527	T	C	176	M	T
	961–963	CCA	–	321	P	–
chrA09g005072	551	G	C	184	G	A
chrA09g005075	47	C	G	16	S	C
	829	A	G	277	S	G
	893	G	A	298	R	K
	1132	A	G	378	I	V
	1188–1189	–	CCTCCT	396–397	–	PP
chrA09g005077	603	C	G	201	D	E
	1253	C	A	418	T	N
chrA09g005091	166	C	T	56	L	F
chrA09g005092	602	G	C	201	G	A

## Discussion

Rapeseed silique plays an important role in the accumulation of photosynthetic products, and it was demonstrated that longer siliques with higher seed density could improve the yield of the final products by increasing seed number directly or indirectly^13,14^. Therefore, creating and selecting new varieties with long silique and high seed density may be an effective strategy to increase the rapeseed yield. Previous studies have shown that most agronomic traits in rapeseed, including those related to seeds and siliques, are complex quantitative traits and are controlled by multiple gene products^4,5,14^. Although many QTL for seed weight, SPS, and VSL and underlying genes have been validated and characterized in rapeseed^5,7,14^, only a few QTL or genes are reported for SDPS, the ratio of SL to SPS, in rapeseed.

QTL-seq that combines BSA and NGS has been successfully used to rapidly identify many QTL for different traits in rapeseed^6,15,16^. In the present study, QTL that controls SDPS was identified on rapeseed chromosome A09 in a mapping population derived from the 4263 × 3001 crosses. Further, two QTL (*qSD.A9-1* and *qSL.A9*) with 53.1% PV for SDPS from one parent (4263) and 47.6% PV for VSL from the other parent (3001) were mapped on chromosome A09 between 42.22 Mb and 42.63 Mb based on Ningyou7 reference genome sequence^12^. Near this genomic position, one minor QTL for SDPS and two minor QTL for SPS were identified. We speculate the existence of a gene or gene cluster in this region that simultaneously regulates SDPS and VSL or SDPS and SPS. Earlier, a QTL integration analysis performed by Zhou et al. (2021)^14^ identified the QTL for SDPS (*cqSDPS-A9-2*), VSL (*cqSL-A9-2*), and SPS (*cqSPS-A9-2*) at the same location on chromosome A09. The physical location of the major QTL associated with VSL and SDPS was detected on chromosome A09 between 27.76 Mb and 28.09 Mb based on ZS11 genome sequence^17^; this overlapped with previously reported QTL (*cqSL-A9-2*)^14^.

To recapitulate the physical location of linkage markers (*Nys9A190* and *Nys9A531*) based on Ningyou7 reference genome^12^, 56 genes were predicted in this region, and 36 of these genes had exonic (splicing) changes. Of all the predicted genes, *ChrA09g005075* and *ChrA09g005049* were the most likely candidate for *qSL.A9* and *qSPDS.A9-1*, respectively. This is because several previous studies have proved that genes associated with auxin repression affect silique length in *Brassica* species, and 2OG-Fe(II) oxygenase is a key player in GA and ABA signal transduction^18,19^. GA plays an important role in regulating seed growth and size, while ABA plays an important role in regulating seed number^18^. Among other important genes, those related to functions of seed development are attractive candidates; however, more studies are required to validate their association with SDPS and VSL.

## Materials and methods

### Plant materials

All seeds of rapeseed accessions used in this study were obtained from the Shanghai Academy of Agricultural Sciences. 4263 had a higher SDPS (more than 3.72 seeds/cm) than that of 3001 (less than 2.24 seeds/cm), while the VSL of 3001 (7.56 cm) was longer than that of 4263 (5.81 cm) in different years. The two rapeseed lines (4263 with short silique but high seed density and 3001 with long silique but low seed density) were selected to develop the mapping population to detect QTL for SDPS and its related traits (Fig. S3). 4263 is a new rapeseed line derived from a self-cross plant of the rapeseed germplasm Su YJ-3, while 3001, which was used as the male, is a new rapeseed line developed from a cross between rapeseed lines Rong Xuan and Jian7. The 4263 × 3001 hybrids were advanced from the F_1_ cross by self-cross to yield the segregation population for identifying the QTL of SDPS and its related traits.

### Plant growth conditions and evaluation of phenotypes

All rapeseed materials including two parental lines, their F_1_ plants, F_2_ individuals, and/or F_2:3_ families were planted in normal growing seasons at the experiment base of Shanghai Academy of Agricultural Sciences, Shanghai, China. Each row contained 13–15 plants with a planting density of 30 cm between rows and 20 cm between plants within each row. All lines were open-pollinated to keep a full record of fertilization rates. The field management was conducted with normal agricultural practices. At the mature stage, 10 well-developed siliques of the main raceme were harvested randomly from each F_2_ plant to analyze the SDPS and its related traits as described by Wang et al^4^. Phenotypic data per plant was collected as follows: (1) VSL (cm): estimated from the average length (excluding the beak length) of all chosen siliques. (2) SPS: estimated from the average value of all chosen siliques. (3) SDPS (seed number/cm): measured using the ratio of VSL to SPS. For each of the F_2:3_ families, the F_3_ individuals with over 20 plants were grown in two rows, which enabled enough plants to evaluate the phenotype of the F_2_ plants. Twenty plants from each F_2:3_ family and parental line were selected for the phenotypic analysis at the mature stage. Subsequently, five well-developed siliques of the main raceme were harvested from each plant to measure SDPS and its related trait as described by Wang et al^4^ and Zhang et al^5^. The phenotypic data of SDPS and its related traits for each F_2:3_ families were collected as a mean from the 20 F_3_ plants, so as two parental lines. The broad-sense heritability was estimated using parent–offspring regression methods based on the variance of parents, F_1_-derived F_2_, and F_2_-derived F_3_ lines^20^.

### Sample bulking and DNA isolation

A total of 780 F_2_ individuals from 4263 × 3001 crosses were selected to build DNA bulks for QTL-seq. Forty individuals each for HT and LT bulks were selected from the F_2_ population based on their extreme phenotypes for SDPS. Genomic DNA from HT bulk, LT bulk, their parents (4263 and 3001), and F_2_ individuals were isolated using Plant Genomic DNA Extraction Kit (TIANGEN, China), following the manufacturer’s instructions. The quality and concentration of DNA samples were examined by agarose gel electrophoresis (1.5%; w/v).

### Illumina sequencing and NGS data analysis

Test-qualified DNA samples from HT bulk, LT bulk, and two parents (4263 and 3001) were used to construct libraries with an insert size of 350–500 bp using the TruSeq DNA LT Sample Prep Kit and sequenced (150 bp pair-end reads) via an Illumina Xten platform at Shanghai OE Biotech Co., Ltd. (China). Raw data produced from Illumina sequencing were subjected to quality control by Trimmomatic (v0.36)^21^. The filtered clean reads from two DNA bulks and their parents were aligned to the Ningyou7 rapeseed genome^12^ using BWA^22^, and SNP calling was performed using SAMtools^23^. The average SNP index was calculated in 1 Mb sliding windows with a 20 kb increment for each pool based on their parent genotype. The △(SNP-index) was calculated by subtracting the SNP-index of LT bulk from that of HT bulk. All SNP-index and △(SNP-index) for all positions were calculated to identify the QTL of SDPS as previously described^6,24^.

### SSR marker analysis and QTL fine mapping

QTL identified by QTL-seq were validated and fine-mapped through linkage analysis in F_2:3_ families. Primers in the predicted regions were designed using SSR Locator^25^ with the parameters as previously described^26^ based on the reference genome^12^. All newly developed markers were named as previously described^15^. PCR and amplicon detection in F_2_ plants and parental lines were performed with minor modifications as previously described^27^. The polymorphic markers were selected to detect the F_2_ population for linkage map construction. Further, the linkage map was drawn using MAP functionality in QTL IciMapping v4.1^28^ with the map distance (cM) of Kosambi mapping function^29^. QTL was conducted using the BIP functionality with the ICIM-ADD mapping method in QTL IciMapping v4.1^28^. The LOD threshold and recombination frequency of QTL mapping were set at 3.0 and 0.30, respectively. The designated QTL for SDPS, VSL, and SP was named *qSD*, *qSL*, and *qSP* followed by the chromosome number, respectively.

### Candidate gene analysis of the major QTL for SDPS and VSL

The candidate genes for the major QTL of SDPS and/or VSL were obtained using the Ningyou7 rapeseed genome annotation^12^. Furthermore, the SNPs or InDels in each gene were predicted using SnpEff^30^.

### Permission statement

All the experiments on plant resources, including the collection of rapeseed germplasms, were performed following relevant local guidelines and regulations.

## Conclusions

In this study, a total of 780 F_2_ lines and 573 F_2:3_ families were constructed to elucidate the genetic mechanisms of seed density and its related traits. One major QTL was mapped to a 401.8 kb interval between *Nys9A190* and *Nys9A531* on rapeseed chromosome A09 and was associated with seed density and silique length in this population based on QTL-seq and linkage analysis. The PV of SDPS and VSL was 53.1% and 47.6%, respectively, in F_2:3_ families. These findings provide a basis to conduct genetic breeding experiments involving cloning of both seed density and silique length in rapeseed.

## Supplementary Information

Supplementary Information 1.

Supplementary Information 2.

Supplementary Information 3.

Supplementary Information 4.

## Data Availability

This whole genome resequencing reads of QTL-seq have been deposited in the National Center of Biotechnology Information Sequence Read Archive (SRA) under BioProject accession number PRJNA885277 (https://www.ncbi.nlm.nih.gov/bioproject/PRJNA885277).
