# Supplementary figures and images for: QTL mapping for seed density per silique in Brassica napus
Source: Sci Rep. 2023 Jan 14;13:772. doi: 10.1038/s41598-023-28066-5 (PMC9840639; doi:10.1038/s41598-023-28066-5)

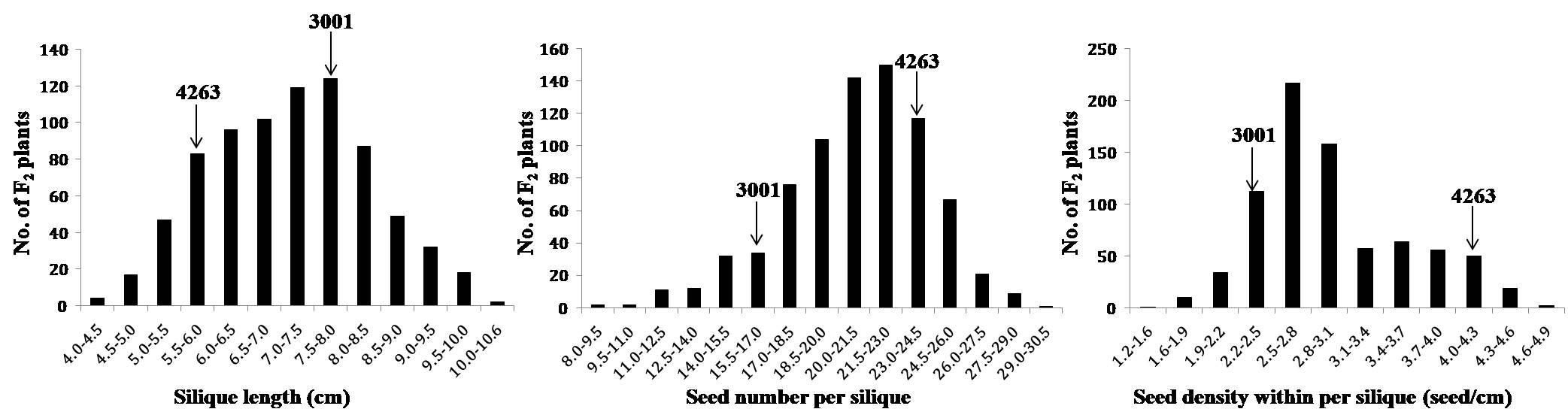

Supplement: Supplementary file 1 — Supplementary Information 1. [file 41598_2023_28066_MOESM1_ESM.jpg]

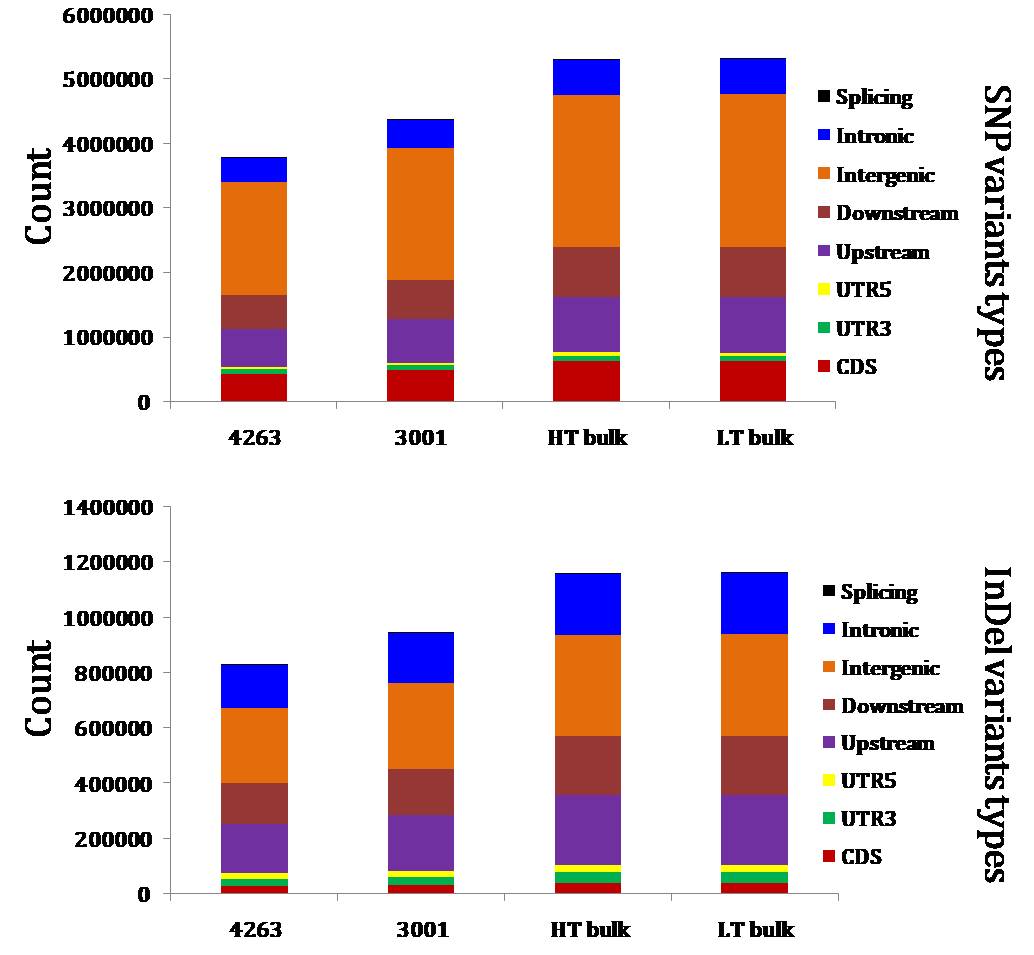

Supplement: Supplementary file 2 — Supplementary Information 2. [file 41598_2023_28066_MOESM2_ESM.jpg]

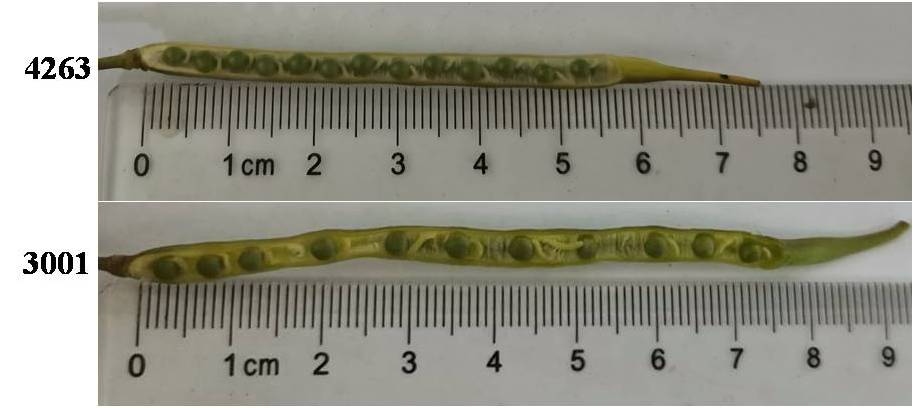

Supplement: Supplementary file 3 — Supplementary Information 3. [file 41598_2023_28066_MOESM3_ESM.jpg]
