# Supplementary material for: QTL mapping for seed density per silique in Brassica napus
Source: Sci Rep. 2023 Jan 14;13:772. doi: 10.1038/s41598-023-28066-5 (PMC9840639; doi:10.1038/s41598-023-28066-5)
Supplement: Supplementary file 4 — Supplementary Information 4. [file 41598_2023_28066_MOESM4_ESM.pdf]

QTL mapping for seed density within per silique in *Brassica napus*

Authors: Jifeng Zhu, Lei Lei, Weirong Wang, Jianxia Jiang, Xirong Zhou*

Key Laboratory of Germplasm innovation and genetic improvement of grain and oil crops (Co-construction by Ministry and Province), Ministry of Agriculture and Rural Affairs, Crop Breeding and Cultivation Research Institute, Shanghai Academy of Agricultural Sciences, Shanghai, 201403, *China*

*Corresponding author: zwrape2021@163.com

Supplementary information

Figure S1. Distribution of the SDPS and its related traits phenotype in F_2 population.

Figure S2. Types of SNP and InDel variants in HT bulk, LT bulk and two parental lines.

Figure S3. The SDPS and its related traits in 4263 and 3301.

Table S1 Number of SNPs and InDels were identified in HT bulk, LT bulk and two parental lines.

Type	HT bulk	LT bulk	4263	3001
Number of SNPs	5,301,640	5,313,578	3,785,071	4,368,424
Number of InDels	1,159,752	1,163,307	830,953	944,643

Table S2 Function variants effects related to the predicted genes in the region of *qSD.A9-1* or/and *qSL.A9*.

Mutation position	The base of 4263	The base of 3001	GeneID	Mutation type
42228851	G	G,A	*ChrA09g005038*	nonsynonymous SNV
42229086	C	A	*ChrA09g005038*	synonymous SNV
42229143	C	A	*ChrA09g005038*	synonymous SNV
42229152	C	T	*ChrA09g005038*	synonymous SNV
42229158	T	C	*ChrA09g005038*	synonymous SNV
42229161	A	C	*ChrA09g005038*	synonymous SNV
42229186	T	G	*ChrA09g005038*	nonsynonymous SNV
42229359	C	T	*ChrA09g005038*	synonymous SNV
42230150	G	T	*ChrA09g005038*	intronic
42230162	G	C	*ChrA09g005038*	intronic
42230166	T	TA	*ChrA09g005038*	intronic
42230190	T	TTA	*ChrA09g005038*	intronic
42230192	G	T	*ChrA09g005038*	intronic
42230195	A	AG	*ChrA09g005038*	intronic
42230208	C	T	*ChrA09g005038*	intronic
42230213	G	A	*ChrA09g005038*	intronic
42230424	G	T	*ChrA09g005038*	UTR3
42230443	G	A	*ChrA09g005038*	UTR3

42230491	T	C	*ChrA09g005038*	UTR3
42230515	ATATTCTGTAGAATAT	A	*ChrA09g005038*	UTR3
42230539	G	A	*ChrA09g005038*	UTR3
42230838	G	A	*ChrA09g005039*	synonymous SNV
42230904	G	A	*ChrA09g005039*	synonymous SNV
42230937	C	G	*ChrA09g005039*	synonymous SNV
42230946	T	C	*ChrA09g005039*	synonymous SNV
42230961	T	C	*ChrA09g005039*	synonymous SNV
42230978	G	A	*ChrA09g005039*	nonsynonymous SNV
42231000	A	G	*ChrA09g005039*	synonymous SNV
42231003	C	T	*ChrA09g005039*	synonymous SNV
42231057	A	G	*ChrA09g005039*	synonymous SNV
42231062	G	C	*ChrA09g005039*	nonsynonymous SNV
42231423	G	C	*ChrA09g005039*	synonymous SNV
42231542	T	C	*ChrA09g005039*	nonsynonymous SNV
42231549	A	C	*ChrA09g005039*	synonymous SNV
42231698	A	G	*ChrA09g005039*	synonymous SNV
42231719	C	T	*ChrA09g005039*	nonsynonymous SNV
42231849	G	A	*ChrA09g005039*	synonymous SNV
42231936	T	C	*ChrA09g005039*	synonymous SNV
42231954	A	G	*ChrA09g005039*	synonymous SNV
42232454	C	CT	*ChrA09g005040*	intronic
42232522	C	A	*ChrA09g005040*	intronic
42232532	G	GA	*ChrA09g005040*	intronic
42232534	T	C	*ChrA09g005040*	intronic
42232549	G	A	*ChrA09g005040*	intronic
42232551	T	G	*ChrA09g005040*	intronic
42232564	G	A	*ChrA09g005040*	intronic
42232845	C	T	*ChrA09g005040*	UTR5
42232875	G	T	*ChrA09g005040*	nonsynonymous SNV
42232908	G	A	*ChrA09g005040*	nonsynonymous SNV
42232909	C	A	*ChrA09g005040*	nonsynonymous SNV
42232928	G	A	*ChrA09g005040*	nonsynonymous SNV
42233214	T	C	*ChrA09g005040*	nonsynonymous SNV
42233230	T	A	*ChrA09g005040*	nonsynonymous SNV
42233280	G	GACA	*ChrA09g005040*	nonframeshift insertion
42233331	G	A	*ChrA09g005040*	UTR3
42233348	T	A	*ChrA09g005040*	UTR3
42233374	T	TA	*ChrA09g005040*	UTR3
42233489	A	G	*ChrA09g005040*	UTR3

42233490	A	G	*ChrA09g005040*	UTR3
42233499	TCA	T	*ChrA09g005040*	UTR3
42233682	G	T	*ChrA09g005041*	UTR3
42233751	TAGAC	T	*ChrA09g005041*	UTR3
42233773	T	G	*ChrA09g005041*	UTR3
42233848	T	C	*ChrA09g005041*	nonsynonymous SNV
42233882	C	T,C	*ChrA09g005041*	synonymous SNV
42233981	A	A,T	*ChrA09g005041*	synonymous SNV
42233990	C	T	*ChrA09g005041*	synonymous SNV
42234119	A	G	*ChrA09g005041*	synonymous SNV
42234140	G	A	*ChrA09g005041*	synonymous SNV
42234145	G	A	*ChrA09g005041*	nonsynonymous SNV
42234149	A	G	*ChrA09g005041*	synonymous SNV
42234155	A	G	*ChrA09g005041*	synonymous SNV
42234926	C	A	*ChrA09g005041*	nonsynonymous SNV
42234974	G	T	*ChrA09g005041*	nonsynonymous SNV
42235372	T	A	*ChrA09g005041*	nonsynonymous SNV
42236926	A	G	*ChrA09g005041*	upstream
42250165	T	C	*ChrA09g005044*	nonsynonymous SNV
42250188	G	T	*ChrA09g005044*	nonsynonymous SNV
42250251	C	CT	*ChrA09g005043*	UTR3
42250714	C	G	*ChrA09g005044*	nonsynonymous SNV
42252895	C	T	*ChrA09g005043*	intronic
42252928	GATT	G	*ChrA09g005043*	intronic
42252993	G	A	*ChrA09g005043*	synonymous SNV
42253077	G	T	*ChrA09g005043*	synonymous SNV
42253231	G	GAAAGA	*ChrA09g005043*	intronic
42253311	CAAAAACAA	C	*ChrA09g005043*	intronic
42253522	A	AATG	*ChrA09g005043*	intronic
42253568	AG	A	*ChrA09g005043*	intronic
42253651	G	G,C	*ChrA09g005043*	UTR5
42253661	TA	TA,T	*ChrA09g005043*	UTR5
42253664	TAA	TAA,T	*ChrA09g005043*	UTR5
42253801	C	G	*ChrA09g005043*	intronic
42253806	G	A	*ChrA09g005043*	intronic
42253969	TA	T	*ChrA09g005043*	UTR5
42253975	C	G	*ChrA09g005043*	UTR5
42254115	A	AG	*ChrA09g005043*	UTR5
42254149	A	AT	*ChrA09g005043*	UTR5
42254150	A	AAT	*ChrA09g005043*	UTR5

42254160	T	TGAGA	*ChrA09g005043*	UTR5
42254174	A	T	*ChrA09g005043*	UTR5
42254186	A	G	*ChrA09g005043*	UTR5
42254200	A	T	*ChrA09g005043*	UTR5
42254226	G	GTTAAAAC	*ChrA09g005043*	UTR5
42258941	T	C	*ChrA09g005045*	intronic
42258951	T	A	*ChrA09g005045*	intronic
42258978	T	C	*ChrA09g005045*	intronic
42258986	T	C	*ChrA09g005045*	intronic
42259016	T	A	*ChrA09g005045*	intronic
42259079	A	G	*ChrA09g005045*	intronic
42259089	G	A	*ChrA09g005045*	intronic
42259131	TG	T	*ChrA09g005045*	intronic
42259138	TTC	T	*ChrA09g005045*	intronic
42260027	G	A	*ChrA09g005045*	UTR3
42260250	T	C	*ChrA09g005045*	downstream
42260257	C	T	*ChrA09g005045*	downstream
42260295	G	A	*ChrA09g005045*	downstream
42260319	G	A	*ChrA09g005045*	downstream
42260374	A	T	*ChrA09g005045*	downstream
42260416	C	T	*ChrA09g005045*	downstream
42260426	C	CCA	*ChrA09g005045*	downstream
42260474	C	T	*ChrA09g005045*	downstream
42260478	T	TAA	*ChrA09g005045*	downstream
42260512	A	AAAGTATAGATAG	*ChrA09g005045*	downstream
42260742	A	G	*ChrA09g005045*	downstream
42260822	A	G	*ChrA09g005045*	downstream
42260879	C	T	*ChrA09g005045*	downstream
42260907	T	C	*ChrA09g005045*	downstream
42261034	G	A	*ChrA09g005045*	downstream
42261098	AT	A	*ChrA09g005045*	downstream
42261801	T	C	*ChrA09g005045*	downstream
42261974	G	GT	*ChrA09g005045*	downstream
42261999	T	C	*ChrA09g005045*	downstream
42262040	C	CT	*ChrA09g005045*	downstream
42262309	A	AT	*ChrA09g005045-ChrA09g005046*	intergenic
42262310	G	T	*ChrA09g005045-ChrA09g005046*	intergenic
42262331	G	A	*ChrA09g005045-ChrA09g005046*	intergenic
42263006	G	A	*ChrA09g005045-ChrA09g005046*	intergenic
42263149	T	A	*ChrA09g005045-ChrA09g005046*	intergenic

42263522	C	C,G	*ChrA09g005046*	upstream
42264841	A	T	*ChrA09g005046*	upstream
42264974	C	T	*ChrA09g005046*	upstream
42265243	A	G	*ChrA09g005046*	upstream
42265418	A	T	*ChrA09g005046*	UTR5
42265498	C	A	*ChrA09g005046*	UTR5
42265504	C	T	*ChrA09g005046*	UTR5
42265521	T	G	*ChrA09g005046*	UTR5
42265530	C	T	*ChrA09g005046*	UTR5
42266100	A	C	*ChrA09g005046*	intronic
42269574	C	G	*ChrA09g005046*	intronic
42269584	T	G	*ChrA09g005046*	intronic
42271032	T	A	*ChrA09g005046*	synonymous SNV
42271058	GA	G	*ChrA09g005046*	UTR3
42271363	G	GA	*ChrA09g005046*	downstream
42271466	C	A	*ChrA09g005046*	downstream
42273688	A	G	*ChrA09g005047*	upstream
42273719	A	G	*ChrA09g005047*	upstream
42273727	A	G	*ChrA09g005047*	upstream
42274751	A	G	*ChrA09g005047*	intronic
42274759	G	T	*ChrA09g005047*	intronic
42274823	G	C	*ChrA09g005047*	synonymous SNV
42274869	T	A	*ChrA09g005047*	intronic
42274877	C	T	*ChrA09g005047*	intronic
42274885	C	T	*ChrA09g005047*	intronic
42275716	A	G	*ChrA09g005047*	downstream
42281899	G	A	*ChrA09g005047-ChrA09g005048*	intergenic
42284702	G	T	*ChrA09g005048*	downstream
42284908	C	A	*ChrA09g005048*	downstream
42284926	T	C	*ChrA09g005048*	downstream
42285041	A	C	*ChrA09g005048*	downstream
42285261	T	T,C	*ChrA09g005048*	downstream
42285298	C	C,T	*ChrA09g005048*	downstream
42286277	G	C	*ChrA09g005048*	synonymous SNV
42286307	A	G	*ChrA09g005048*	synonymous SNV
42286492	G	A	*ChrA09g005048*	nonsynonymous SNV
42286579	GAGA	G	*ChrA09g005048*	UTR5
42286701	A	G	*ChrA09g005048*	UTR5
42286712	G	A	*ChrA09g005048*	UTR5
42286792	G	A	*ChrA09g005048*	UTR5

42287041	G	C	*ChrA09g005048*	upstream
42288674	CG	C	*ChrA09g005048*	upstream
42289585	T	A	*ChrA09g005048-ChrA09g005049*	intergenic
42289635	A	C	*ChrA09g005048-ChrA09g005049*	intergenic
42290607	A	T	*ChrA09g005049*	downstream
42290916	G	GCGTTATAGTTTAAACATTTTTTGAAACT	*ChrA09g005049*	downstream
42291408	GTATATATA	G	*ChrA09g005049*	downstream
42291548	A	C	*ChrA09g005049*	downstream
42291559	A	AT	*ChrA09g005049*	downstream
42291561	A	AAAT	*ChrA09g005049*	downstream
42291885	C	G	*ChrA09g005049*	nonsynonymous SNV
42292252	C	T	*ChrA09g005049*	nonsynonymous SNV
42294275	T	A	*ChrA09g005049*	nonsynonymous SNV
42294297	C	T	*ChrA09g005049*	synonymous SNV
42294523	T	G	*ChrA09g005049*	nonsynonymous SNV
42295781	T	TAG	*ChrA09g005050*	UTR5
42295950	G	A	*ChrA09g005050*	synonymous SNV
42296162	C	T	*ChrA09g005050*	synonymous SNV
42296165	C	T	*ChrA09g005050*	synonymous SNV
42296214	C	T	*ChrA09g005050*	intronic
42296514	G	C	*ChrA09g005050*	intronic
42296593	T	C	*ChrA09g005050*	intronic
42296701	A	AT	*ChrA09g005050*	intronic
42304341	G	A	*ChrA09g005051*	UTR5
42304479	A	G	*ChrA09g005051*	UTR5
42304612	C	T	*ChrA09g005051*	nonsynonymous SNV
42304901	T	C	*ChrA09g005051*	synonymous SNV
42304971	T	C	*ChrA09g005051*	synonymous SNV
42304976	T	C	*ChrA09g005051*	synonymous SNV
42304997	T	C	*ChrA09g005051*	synonymous SNV
42305026	C	T	*ChrA09g005051*	nonsynonymous SNV
42305033	A	T	*ChrA09g005051*	synonymous SNV
42305242	G	A	*ChrA09g005051*	nonsynonymous SNV
42305310	T	C	*ChrA09g005051*	synonymous SNV
42305321	G	C	*ChrA09g005051*	synonymous SNV
42305417	T	A	*ChrA09g005051*	intronic
42305425	T	C	*ChrA09g005051*	intronic
42305709	G	T	*ChrA09g005051*	intronic
42305716	G	A	*ChrA09g005051*	intronic
42305721	A	G	*ChrA09g005051*	intronic

42305732	A	C	*ChrA09g005051*	intronic
42305735	A	G	*ChrA09g005051*	intronic
42305736	T	A	*ChrA09g005051*	intronic
42305745	TA	T	*ChrA09g005051*	intronic
42305747	A	G	*ChrA09g005051*	intronic
42305749	A	T	*ChrA09g005051*	intronic
42305753	A	T	*ChrA09g005051*	intronic
42305758	A	G	*ChrA09g005051*	intronic
42305759	AAG	A	*ChrA09g005051*	intronic
42305763	T	C	*ChrA09g005051*	intronic
42305770	T	G	*ChrA09g005051*	intronic
42305772	TA	T	*ChrA09g005051*	intronic
42305776	T	G	*ChrA09g005051*	intronic
42305781	A	T	*ChrA09g005051*	intronic
42305789	A	G	*ChrA09g005051*	intronic
42305802	AT	A	*ChrA09g005051*	intronic
42305808	T	A	*ChrA09g005051*	intronic
42305813	TA	T	*ChrA09g005051*	intronic
42305816	TCAAAC	T	*ChrA09g005051*	intronic
42305823	A	T	*ChrA09g005051*	intronic
42305828	T	TTCAGGG	*ChrA09g005051*	intronic
42305843	C	CG	*ChrA09g005051*	intronic
42305847	A	T	*ChrA09g005051*	intronic
42305848	A	T	*ChrA09g005051*	intronic
42305858	A	G	*ChrA09g005051*	intronic
42305874	T	TA	*ChrA09g005051*	intronic
42306193	T	TCGC	*ChrA09g005051*	intronic
42306197	C	CG	*ChrA09g005051*	intronic
42306198	A	C	*ChrA09g005051*	intronic
42306207	T	C	*ChrA09g005051*	intronic
42306270	C	G	*ChrA09g005051*	synonymous SNV
42306288	T	C	*ChrA09g005051*	synonymous SNV
42306354	T	C	*ChrA09g005051*	synonymous SNV
42306366	C	G	*ChrA09g005051*	synonymous SNV
42306417	G	C	*ChrA09g005051*	synonymous SNV
42306450	A	G	*ChrA09g005051*	synonymous SNV
42306498	G	C	*ChrA09g005051*	synonymous SNV
42306874	GAGA	G	*ChrA09g005051*	nonframeshift deletion
42307122	T	C	*ChrA09g005051*	downstream
42307169	GCTTGCAAGATGTGCCGATATAGAGC	G	*ChrA09g005051*	downstream

42307268	T	C	*ChrA09g005051*	downstream
42307586	G	T	*ChrA09g005051*	downstream
42307729	G	GTAC	*ChrA09g005051*	downstream
42313195	C	CTGA	*ChrA09g005051-ChrA09g005052*	intergenic
42313200	GGT	G	*ChrA09g005051-ChrA09g005052*	intergenic
42313386	A	G	*ChrA09g005051-ChrA09g005052*	intergenic
42313394	G	GT	*ChrA09g005051-ChrA09g005052*	intergenic
42313580	C	G	*ChrA09g005051-ChrA09g005052*	intergenic
42313596	G	A	*ChrA09g005051-ChrA09g005052*	intergenic
42313610	A	G	*ChrA09g005051-ChrA09g005052*	intergenic
42313616	G	T	*ChrA09g005051-ChrA09g005052*	intergenic
42313637	G	A	*ChrA09g005051-ChrA09g005052*	intergenic
42313644	G	A	*ChrA09g005051-ChrA09g005052*	intergenic
42313645	G	A	*ChrA09g005051-ChrA09g005052*	intergenic
42313654	T	G	*ChrA09g005051-ChrA09g005052*	intergenic
42313685	A	G	*ChrA09g005051-ChrA09g005052*	intergenic
42313701	TTGGGTTTAG	T	*ChrA09g005051-ChrA09g005052*	intergenic
42313712	ATTTAG	A	*ChrA09g005051-ChrA09g005052*	intergenic
42313734	A	T	*ChrA09g005051-ChrA09g005052*	intergenic
42313758	A	G	*ChrA09g005051-ChrA09g005052*	intergenic
42313760	A	ATTTAGGG	*ChrA09g005051-ChrA09g005052*	intergenic
42313778	T	C	*ChrA09g005051-ChrA09g005052*	intergenic
42313806	A	ATT	*ChrA09g005051-ChrA09g005052*	intergenic
42313817	AAT	A	*ChrA09g005051-ChrA09g005052*	intergenic
42313846	C	T	*ChrA09g005051-ChrA09g005052*	intergenic
42313850	A	G	*ChrA09g005051-ChrA09g005052*	intergenic
42313867	G	C	*ChrA09g005051-ChrA09g005052*	intergenic
42313870	T	A	*ChrA09g005051-ChrA09g005052*	intergenic
42313890	CA	C	*ChrA09g005051-ChrA09g005052*	intergenic
42313894	T	C	*ChrA09g005051-ChrA09g005052*	intergenic
42313896	AG	A	*ChrA09g005051-ChrA09g005052*	intergenic
42313904	A	G	*ChrA09g005051-ChrA09g005052*	intergenic
42313910	G	A	*ChrA09g005051-ChrA09g005052*	intergenic
42313924	C	T	*ChrA09g005051-ChrA09g005052*	intergenic
42313932	A	G	*ChrA09g005051-ChrA09g005052*	intergenic
42313933	C	G	*ChrA09g005051-ChrA09g005052*	intergenic
42313938	G	GCA	*ChrA09g005051-ChrA09g005052*	intergenic
42313942	AGG	A	*ChrA09g005051-ChrA09g005052*	intergenic
42313946	T	C	*ChrA09g005051-ChrA09g005052*	intergenic
42313987	C	A	*ChrA09g005051-ChrA09g005052*	intergenic

42313997	T	A	*ChrA09g005051-ChrA09g005052*	intergenic
42314007	CAA	C	*ChrA09g005051-ChrA09g005052*	intergenic
42314015	A	AGGGC	*ChrA09g005051-ChrA09g005052*	intergenic
42314072	T	G	*ChrA09g005051-ChrA09g005052*	intergenic
42314075	C	G	*ChrA09g005051-ChrA09g005052*	intergenic
42314081	T	C	*ChrA09g005051-ChrA09g005052*	intergenic
42314111	G	A	*ChrA09g005051-ChrA09g005052*	intergenic
42314115	T	G	*ChrA09g005051-ChrA09g005052*	intergenic
42314121	G	A	*ChrA09g005051-ChrA09g005052*	intergenic
42314128	G	C	*ChrA09g005051-ChrA09g005052*	intergenic
42314134	G	A	*ChrA09g005051-ChrA09g005052*	intergenic
42314161	T	G	*ChrA09g005051-ChrA09g005052*	intergenic
42314194	C	T	*ChrA09g005051-ChrA09g005052*	intergenic
42314227	G	A	*ChrA09g005051-ChrA09g005052*	intergenic
42314265	G	G,A	*ChrA09g005051-ChrA09g005052*	intergenic
42314269	G	A	*ChrA09g005051-ChrA09g005052*	intergenic
42314270	C	C,T	*ChrA09g005051-ChrA09g005052*	intergenic
42314271	G	G,T	*ChrA09g005051-ChrA09g005052*	intergenic
42314813	AATAT	A	*ChrA09g005051-ChrA09g005052*	intergenic
42314859	C	T	*ChrA09g005051-ChrA09g005052*	intergenic
42315008	A	G	*ChrA09g005051-ChrA09g005052*	intergenic
42315009	C	T	*ChrA09g005051-ChrA09g005052*	intergenic
42315022	G	A	*ChrA09g005051-ChrA09g005052*	intergenic
42315033	C	T	*ChrA09g005051-ChrA09g005052*	intergenic
42315044	G	A	*ChrA09g005051-ChrA09g005052*	intergenic
42315072	T	C	*ChrA09g005051-ChrA09g005052*	intergenic
42315192	G	A	*ChrA09g005051-ChrA09g005052*	intergenic
42317278	G,T	T	*ChrA09g005052*	upstream
42317885	A	A,AT	*ChrA09g005052*	intronic
42318185	G	C	*ChrA09g005052*	nonsynonymous SNV
42319292	C	T	*ChrA09g005052*	intronic
42319556	C	T	*ChrA09g005052*	nonsynonymous SNV
42320663	T	A	*ChrA09g005053*	intronic
42320838	T	TCAA	*ChrA09g005053*	intronic
42320889	C	A	*ChrA09g005053*	intronic
42321004	A	C	*ChrA09g005053*	intronic
42321010	A	G	*ChrA09g005053*	intronic
42321227	A	C	*ChrA09g005053*	intronic
42321583	A	G	*ChrA09g005053*	nonsynonymous SNV
42322968	C	G	*ChrA09g005054*	synonymous SNV

42323076	C	T	*ChrA09g005054*	synonymous SNV
42323079	A	C	*ChrA09g005054*	synonymous SNV
42323085	C	T	*ChrA09g005054*	synonymous SNV
42323106	G	A	*ChrA09g005054*	synonymous SNV
42323755	G	T	*ChrA09g005054*	intronic
42323897	G	C	*ChrA09g005054*	intronic
42323911	CT	C	*ChrA09g005054*	intronic
42324465	C	G	*ChrA09g005054*	synonymous SNV
42324555	T	TTA	*ChrA09g005054*	intronic
42324667	G	C	*ChrA09g005054*	synonymous SNV
42324768	C	T	*ChrA09g005054*	upstream
42324791	T	A	*ChrA09g005054*	upstream
42324809	ATAT	A	*ChrA09g005054*	upstream
42324834	A	G	*ChrA09g005054*	upstream
42324953	G	GTT	*ChrA09g005054*	upstream
42325025	C	CAG	*ChrA09g005054*	upstream
42325055	A	C	*ChrA09g005054*	upstream
42325145	T	C	*ChrA09g005054*	upstream
42325207	C	A	*ChrA09g005054*	upstream
42325213	AATAT	A	*ChrA09g005054*	upstream
42325219	T	C	*ChrA09g005054*	upstream
42325277	C	T	*ChrA09g005054*	upstream
42325278	A	T	*ChrA09g005054*	upstream
42325282	A	AAAAACATTTTGAAATTT	*ChrA09g005054*	upstream
42325325	A	C	*ChrA09g005054*	upstream
42325333	AACT	A	*ChrA09g005054*	upstream
42325412	A	G	*ChrA09g005054*	upstream
42325477	T	A	*ChrA09g005054*	upstream
42325552	G	T	*ChrA09g005054*	upstream
42325772	A	G	*ChrA09g005054*	upstream
42325810	G	A	*ChrA09g005054*	upstream
42327132	AGTTTG	A	*ChrA09g005054-ChrA09g005055*	intergenic
42327138	T	TA	*ChrA09g005054-ChrA09g005055*	intergenic
42331656	G	A	*ChrA09g005055*	synonymous SNV
42332059	A	G	*ChrA09g005055*	synonymous SNV
42332110	T	G	*ChrA09g005055*	intronic
42332118	T	TC	*ChrA09g005055*	intronic
42332119	T	A	*ChrA09g005055*	intronic
42332159	A	G	*ChrA09g005055*	intronic
42332699	T	G	*ChrA09g005055*	nonsynonymous SNV

42332758	C	T	*ChrA09g005055*	synonymous SNV
42334843	T	C	*ChrA09g005056*	nonsynonymous SNV
42335124	T	C	*ChrA09g005056*	synonymous SNV
42335223	T	A	*ChrA09g005056*	synonymous SNV
42335275	C	A	*ChrA09g005056*	synonymous SNV
42335325	C	T	*ChrA09g005056*	synonymous SNV
42335373	T	A	*ChrA09g005056*	synonymous SNV
42335391	G	C	*ChrA09g005056*	synonymous SNV
42335394	A	T	*ChrA09g005056*	synonymous SNV
42335437	C	T	*ChrA09g005056*	synonymous SNV
42335570	G	GCTTTA	*ChrA09g005056*	UTR3
42339072	T	G	*ChrA09g005057*	UTR3
42339638	G	A	*ChrA09g005057*	synonymous SNV
42339762	CT	C	*ChrA09g005057*	intronic
42339804	TA	T	*ChrA09g005057*	intronic
42339806	T	G	*ChrA09g005057*	intronic
42340253	A	A,G	*ChrA09g005057*	synonymous SNV
42340257	G,A	A	*ChrA09g005057*	synonymous SNV
42340274	T	G	*ChrA09g005057*	nonsynonymous SNV
42341312	A	G	*ChrA09g005057*	intronic
42341709	CA	C	*ChrA09g005057*	intronic
42341873	G	A	*ChrA09g005057*	intronic
42341882	TG	T	*ChrA09g005057*	intronic
42341904	G	A	*ChrA09g005057*	intronic
42341924	T	TA	*ChrA09g005057*	intronic
42341951	C	T	*ChrA09g005057*	intronic
42341952	C	CAA	*ChrA09g005057*	intronic
42341960	TG	T	*ChrA09g005057*	intronic
42341974	T	G	*ChrA09g005057*	intronic
42341988	C	T	*ChrA09g005057*	intronic
42342045	A	C	*ChrA09g005057*	intronic
42342068	G	T	*ChrA09g005057*	intronic
42342386	A,T	T	*ChrA09g005057*	intronic
42342425	TA	T	*ChrA09g005057*	intronic
42342486	G	A	*ChrA09g005057*	intronic
42342564	A	T	*ChrA09g005057*	synonymous SNV
42342632	C	T	*ChrA09g005057*	intronic
42344388	T	TA	*ChrA09g005058*	UTR5
42344393	A	T	*ChrA09g005058*	UTR5
42344403	GA	G	*ChrA09g005058*	UTR5

42344496	AAG	A	*ChrA09g005058*	UTR5
42344517	T	G	*ChrA09g005058*	UTR5
42344519	GA	G	*ChrA09g005058*	UTR5
42344609	T	C	*ChrA09g005058*	synonymous SNV
42344678	A	C	*ChrA09g005058*	nonsynonymous SNV
42344681	T	C	*ChrA09g005058*	synonymous SNV
42344714	T	C	*ChrA09g005058*	synonymous SNV
42344781	G	A	*ChrA09g005058*	nonsynonymous SNV
42344805	A	G	*ChrA09g005058*	nonsynonymous SNV
42344885	C	A	*ChrA09g005058*	synonymous SNV
42345158	T	C	*ChrA09g005058*	synonymous SNV
42345162	T	C	*ChrA09g005058*	nonsynonymous SNV
42345224	A	C	*ChrA09g005058*	synonymous SNV
42345227	C	A	*ChrA09g005058*	synonymous SNV
42345304	C	G	*ChrA09g005058*	nonsynonymous SNV
42345326	T	C	*ChrA09g005058*	synonymous SNV
42345329	C	G	*ChrA09g005058*	synonymous SNV
42345359	T	C	*ChrA09g005058*	synonymous SNV
42345365	G	A	*ChrA09g005058*	synonymous SNV
42345380	G	A	*ChrA09g005058*	synonymous SNV
42345428	C	C,A	*ChrA09g005058*	synonymous SNV
42345536	C	A	*ChrA09g005058*	synonymous SNV
42345548	A	T	*ChrA09g005058*	synonymous SNV
42345569	G	T	*ChrA09g005058*	synonymous SNV
42345578	T	C	*ChrA09g005058*	synonymous SNV
42345647	T	C	*ChrA09g005058*	synonymous SNV
42345683	C	G	*ChrA09g005058*	synonymous SNV
42345689	C	T	*ChrA09g005058*	synonymous SNV
42345698	C	T	*ChrA09g005058*	synonymous SNV
42345716	T	C	*ChrA09g005058*	synonymous SNV
42345737	T	C	*ChrA09g005058*	synonymous SNV
42345921	T,A	A	*ChrA09g005058*	UTR3
42346377	C	T	*ChrA09g005058*	downstream
42346443	CT	C	*ChrA09g005058*	downstream
42346469	G	A	*ChrA09g005058*	downstream
42346485	T	TTAG	*ChrA09g005058*	downstream
42346519	T	A	*ChrA09g005058*	downstream
42346522	G	A	*ChrA09g005058*	downstream
42347109	TAA	T	*ChrA09g005058*	downstream
42347272	G	G,A	*ChrA09g005058*	downstream

42347540	A	AATT	*ChrA09g005058*	downstream
42347568	T	TAA	*ChrA09g005058*	downstream
42347569	C	A	*ChrA09g005058*	downstream
42347604	T	C	*ChrA09g005058*	downstream
42347611	T	C	*ChrA09g005058*	downstream
42347640	C	T	*ChrA09g005058*	downstream
42347655	T	G	*ChrA09g005058*	downstream
42347677	T	C	*ChrA09g005058*	downstream
42347678	AT	A	*ChrA09g005058*	downstream
42347780	G	A	*ChrA09g005058*	downstream
42347850	C	T	*ChrA09g005058*	downstream
42347851	A	C	*ChrA09g005058*	downstream
42347887	A	G	*ChrA09g005058*	downstream
42348387	AG	A	*ChrA09g005058-ChrA09g005059*	intergenic
42348392	T	A	*ChrA09g005058-ChrA09g005059*	intergenic
42350406	C	T	*ChrA09g005059*	upstream
42350431	C	A	*ChrA09g005059*	upstream
42350468	T	C	*ChrA09g005059*	upstream
42350477	A	G	*ChrA09g005059*	upstream
42350512	G	T	*ChrA09g005059*	upstream
42350550	T	C	*ChrA09g005059*	upstream
42350680	G	T	*ChrA09g005059*	upstream
42350764	G	T	*ChrA09g005059*	UTR5
42350780	A	T	*ChrA09g005059*	UTR5
42350784	C	CT	*ChrA09g005059*	UTR5
42350786	C	CTTCTCTCT	*ChrA09g005059*	UTR5
42350803	TC	T	*ChrA09g005059*	UTR5
42350806	TCTCG	T	*ChrA09g005059*	UTR5
42350817	G	A	*ChrA09g005059*	UTR5
42350820	C	A	*ChrA09g005059*	UTR5
42350909	CT	C	*ChrA09g005059*	UTR5
42350914	T	C	*ChrA09g005059*	UTR5
42350924	TTAA	T	*ChrA09g005059*	UTR5
42350936	A	ATT	*ChrA09g005059*	UTR5
42351108	A	G	*ChrA09g005059*	UTR5
42351109	G	A	*ChrA09g005059*	UTR5
42351118	G	T	*ChrA09g005059*	UTR5
42351328	G	T	*ChrA09g005059*	intronic
42351433	A	AAAGCATGACAC	*ChrA09g005059*	intronic
42351451	A	T	*ChrA09g005059*	intronic

42351467	T	A	*ChrA09g005059*	intronic
42351481	G	T	*ChrA09g005059*	intronic
42351482	GTAC	G	*ChrA09g005059*	intronic
42351526	A	AC	*ChrA09g005059*	intronic
42351532	G	T	*ChrA09g005059*	intronic
42351642	GTTTTGTTTT	G	*ChrA09g005059*	intronic
42351670	A	G	*ChrA09g005059*	UTR5
42351691	A	T	*ChrA09g005059*	synonymous SNV
42351718	T	C	*ChrA09g005059*	synonymous SNV
42351826	C	T	*ChrA09g005059*	synonymous SNV
42351901	C	T	*ChrA09g005059*	synonymous SNV
42351988	C	A	*ChrA09g005059*	nonsynonymous SNV
42352039	C	T	*ChrA09g005059*	synonymous SNV
42352072	C	T	*ChrA09g005059*	synonymous SNV
42352087	G	A	*ChrA09g005059*	synonymous SNV
42352141	T	C	*ChrA09g005059*	synonymous SNV
42352153	G	A	*ChrA09g005059*	synonymous SNV
42352200	T	C	*ChrA09g005059*	nonsynonymous SNV
42352237	C	T	*ChrA09g005059*	synonymous SNV
42352507	A	G	*ChrA09g005059*	synonymous SNV
42352633	AGGT	A	*ChrA09g005059*	nonframeshift deletion
42352708	T	G	*ChrA09g005059*	synonymous SNV
42353030	G	T	*ChrA09g005059*	splicing
42353058	C	T	*ChrA09g005059*	UTR3
42355148	A	G	*ChrA09g005060*	nonsynonymous SNV
42355361	A	C	*ChrA09g005060*	nonsynonymous SNV
42355369	T	A	*ChrA09g005060*	synonymous SNV
42355474	C	T	*ChrA09g005060*	synonymous SNV
42358511	C	T	*ChrA09g005061*	upstream
42358515	A	G	*ChrA09g005061*	upstream
42358566	T	A	*ChrA09g005061*	upstream
42358668	G	A	*ChrA09g005061*	upstream
42358728	T	TA	*ChrA09g005061*	upstream
42358731	G	A	*ChrA09g005061*	upstream
42358774	T	A	*ChrA09g005061*	upstream
42358930	A	C	*ChrA09g005061*	upstream
42358954	T	C	*ChrA09g005061*	upstream
42358960	A	C	*ChrA09g005061*	upstream
42358982	A	G	*ChrA09g005061*	upstream
42358990	T	C	*ChrA09g005061*	upstream

42358993	G	A	*ChrA09g005061*	upstream
42359078	C	G	*ChrA09g005061*	upstream
42359143	T	A	*ChrA09g005061*	upstream
42359197	T	G	*ChrA09g005061*	synonymous SNV
42359323	T	G	*ChrA09g005061*	synonymous SNV
42359350	C	T	*ChrA09g005061*	synonymous SNV
42359421	C	T	*ChrA09g005061*	intronic
42359455	G	T	*ChrA09g005061*	intronic
42359461	A	G	*ChrA09g005061*	intronic
42359621	CT	C	*ChrA09g005061*	intronic
42359858	ATGTGTG	A,ATGTG	*ChrA09g005061*	intronic
42360522	T	TTG	*ChrA09g005061*	intronic
42360601	A	C	*ChrA09g005061*	nonsynonymous SNV
42360863	ATT	A	*ChrA09g005061*	intronic
42360890	C	CT	*ChrA09g005061*	intronic
42361060	C	T	*ChrA09g005061*	synonymous SNV
42361145	A	C	*ChrA09g005061*	nonsynonymous SNV
42361304	T	C	*ChrA09g005061*	nonsynonymous SNV
42361305	T	A	*ChrA09g005061*	nonsynonymous SNV
42361311	C	T	*ChrA09g005061*	nonsynonymous SNV
42361559	G	A	*ChrA09g005061*	nonsynonymous SNV
42364769	GAA	G	*ChrA09g005063*	upstream
42364857	C	A	*ChrA09g005063*	UTR5
42364864	C	T	*ChrA09g005063*	UTR5
42364878	A	T	*ChrA09g005063*	UTR5
42364919	A	T	*ChrA09g005063*	UTR5
42364920	A	G	*ChrA09g005063*	UTR5
42365028	C	T	*ChrA09g005063*	synonymous SNV
42365108	A	G	*ChrA09g005063*	intronic
42365121	T	C	*ChrA09g005063*	intronic
42365123	G	T	*ChrA09g005063*	intronic
42365454	T	G	*ChrA09g005063*	synonymous SNV
42365775	C	T	*ChrA09g005063*	synonymous SNV
42369722	G	C	*ChrA09g005064*	upstream
42383016	A	AAC	*ChrA09g005067*	intronic
42383026	GTT	G	*ChrA09g005067*	intronic
42383037	G	C	*ChrA09g005067*	intronic
42383045	A	AGC	*ChrA09g005067*	intronic
42383048	G	A	*ChrA09g005067*	intronic
42383054	A	AT	*ChrA09g005067*	intronic

42383055	A	ACATGTTGC	*ChrA09g005067*	intronic
42383057	GAGA	G	*ChrA09g005067*	intronic
42385841	T	A	*ChrA09g005068*	nonsynonymous SNV
42385878	T	C	*ChrA09g005068*	nonsynonymous SNV
42385950	T	A	*ChrA09g005068*	nonsynonymous SNV
42386007	A	T	*ChrA09g005068*	nonsynonymous SNV
42386054	T	C	*ChrA09g005068*	nonsynonymous SNV
42386059	A	C	*ChrA09g005068*	synonymous SNV
42386102	T	TAA	*ChrA09g005068*	UTR3
42386104	C	A	*ChrA09g005068*	UTR3
42386675	T	G	*ChrA09g005068*	downstream
42386686	T	C	*ChrA09g005068*	downstream
42386688	A	T	*ChrA09g005068*	downstream
42386702	T	G	*ChrA09g005068*	downstream
42386797	T	G	*ChrA09g005068*	downstream
42386864	G	A	*ChrA09g005068*	downstream
42386926	GTA	G	*ChrA09g005068*	downstream
42388346	G	GTTAGTAC	*ChrA09g005069*	upstream
42388371	T	C	*ChrA09g005069*	upstream
42388385	AT	A	*ChrA09g005069*	upstream
42388584	T	A	*ChrA09g005069*	upstream
42388591	C	A	*ChrA09g005069*	upstream
42388598	T	G	*ChrA09g005069*	upstream
42388605	T	G	*ChrA09g005069*	upstream
42388609	C	T	*ChrA09g005069*	upstream
42388830	A	T	*ChrA09g005069*	upstream
42388841	T	G	*ChrA09g005069*	upstream
42388858	C	G	*ChrA09g005069*	upstream
42389082	TA	T	*ChrA09g005069*	UTR5
42389110	AG	A	*ChrA09g005069*	UTR5
42389115	G	A	*ChrA09g005069*	UTR5
42389152	C	G	*ChrA09g005069*	UTR5
42389154	GAA	G	*ChrA09g005069*	UTR5
42389194	A	C	*ChrA09g005069*	UTR5
42390716	A	AGT	*ChrA09g005069*	frameshift insertion
42390718	CA	C	*ChrA09g005069*	frameshift deletion
42390720	TA	T	*ChrA09g005069*	frameshift deletion
42390887	C	T	*ChrA09g005069*	synonymous SNV
42391055	A	G	*ChrA09g005069*	synonymous SNV
42391325	A	G	*ChrA09g005069*	synonymous SNV

42391379	G	A	*ChrA09g005069*	synonymous SNV
42391490	C	T	*ChrA09g005069*	synonymous SNV
42391499	T	C	*ChrA09g005069*	synonymous SNV
42391589	C	T	*ChrA09g005069*	synonymous SNV
42391625	C	T	*ChrA09g005069*	synonymous SNV
42392085	G	T	*ChrA09g005069*	synonymous SNV
42392565	TTTA	T	*ChrA09g005069*	UTR3
42392571	G	A	*ChrA09g005069*	UTR3
42392610	T	C	*ChrA09g005069*	UTR3
42394463	CTTTTTCTTTCTTTTTCTTTCTT	C	*ChrA09g005069*	downstream
42394634	T	G	*ChrA09g005069*	downstream
42395796	C	A	*ChrA09g005069-ChrA09g005070*	intergenic
42395860	CT	C	*ChrA09g005069-ChrA09g005070*	intergenic
42396357	C	T	*ChrA09g005070*	upstream
42396425	A	G	*ChrA09g005070*	upstream
42396463	G	T	*ChrA09g005070*	upstream
42397384	T	C	*ChrA09g005070*	upstream
42398009	ATTTGTTAAAAAAAAATAAG	A	*ChrA09g005070*	upstream
42398054	TTG	T	*ChrA09g005070*	upstream
42398058	G	T	*ChrA09g005070*	upstream
42398062	GTTTGAGAATC	G	*ChrA09g005070*	upstream
42398116	C	T	*ChrA09g005070*	upstream
42398145	TAAAAAGTTAAAAA	T	*ChrA09g005070*	upstream
42398250	T	C	*ChrA09g005070*	UTR5
42398294	TTCTCTC	T	*ChrA09g005070*	UTR5
42398363	G	GTCCC	*ChrA09g005070*	UTR5
42398410	C	G	*ChrA09g005070*	UTR5
42398444	T	C	*ChrA09g005070*	UTR5
42398480	CGA	C	*ChrA09g005070*	UTR5
42398485	CTTA	C	*ChrA09g005070*	UTR5
42398521	A	G	*ChrA09g005070*	UTR5
42398730	C	T	*ChrA09g005070*	synonymous SNV
42398971	T	C	*ChrA09g005070*	synonymous SNV
42398977	G	A	*ChrA09g005070*	synonymous SNV
42400174	G	T	*ChrA09g005070*	intronic
42400946	C	A	*ChrA09g005070*	nonsynonymous SNV
42401223	C	A	*ChrA09g005070*	nonsynonymous SNV
42401518	A	G	*ChrA09g005070*	nonsynonymous SNV
42401532	G	C	*ChrA09g005070*	nonsynonymous SNV
42401616	G	A	*ChrA09g005070*	nonsynonymous SNV

42401669	G	A	*ChrA09g005070*	synonymous SNV
42401798	T	C	*ChrA09g005070*	synonymous SNV
42401922	T	G	*ChrA09g005070*	nonsynonymous SNV
42402217	G	A	*ChrA09g005070*	nonsynonymous SNV
42403033	G	A	*ChrA09g005070*	synonymous SNV
42404129	A	C	*ChrA09g005070*	downstream
42406095	T	C	*ChrA09g005070-ChrA09g005071*	intergenic
42406176	A	G	*ChrA09g005070-ChrA09g005071*	intergenic
42406343	GA	G	*ChrA09g005070-ChrA09g005071*	intergenic
42406357	A	C	*ChrA09g005070-ChrA09g005071*	intergenic
42406451	TACATGATTG	T	*ChrA09g005070-ChrA09g005071*	intergenic
42406528	C	T	*ChrA09g005070-ChrA09g005071*	intergenic
42407130	G	C	*ChrA09g005070-ChrA09g005071*	intergenic
42407154	T	A	*ChrA09g005070-ChrA09g005071*	intergenic
42407168	ATATATATGTATATATT	A	*ChrA09g005070-ChrA09g005071*	intergenic
42407759	C	T	*ChrA09g005070-ChrA09g005071*	intergenic
42408306	A	T	*ChrA09g005070-ChrA09g005071*	intergenic
42408760	C	T	*ChrA09g005070-ChrA09g005071*	intergenic
42408778	G	GC	*ChrA09g005070-ChrA09g005071*	intergenic
42408806	T	A	*ChrA09g005070-ChrA09g005071*	intergenic
42413003	G	C	*ChrA09g005071*	downstream
42413258	T	C	*ChrA09g005071*	downstream
42413315	C	A	*ChrA09g005071*	downstream
42413731	T	C	*ChrA09g005071*	synonymous SNV
42414133	G	A	*ChrA09g005071*	synonymous SNV
42414445	G	T	*ChrA09g005071*	synonymous SNV
42414489	T	TA	*ChrA09g005071*	intronic
42414495	T	A	*ChrA09g005071*	intronic
42414534	GT	G	*ChrA09g005071*	intronic
42414545	G	GAT	*ChrA09g005071*	intronic
42414546	G	GT	*ChrA09g005071*	intronic
42414602	G	C	*ChrA09g005071*	nonsynonymous SNV
42414993	G	T	*ChrA09g005071*	synonymous SNV
42414996	G	A	*ChrA09g005071*	synonymous SNV
42415257	A	G	*ChrA09g005071*	synonymous SNV
42415978	T	G	*ChrA09g005071*	nonsynonymous SNV
42415979	T	A	*ChrA09g005071*	nonsynonymous SNV
42417339	C	T	*ChrA09g005072*	intronic
42417687	C	G	*ChrA09g005072*	nonsynonymous SNV
42417827	G	A	*ChrA09g005072*	synonymous SNV

42419581	G	A	*ChrA09g005073*	synonymous SNV
42419602	A	G	*ChrA09g005073*	synonymous SNV
42419872	TTATA	T	*ChrA09g005073*	intronic
42419878	A	G	*ChrA09g005073*	intronic
42419950	T	A	*ChrA09g005073*	synonymous SNV
42419953	G	A	*ChrA09g005073*	synonymous SNV
42420045	T	C	*ChrA09g005073*	nonsynonymous SNV
42420109	C	A	*ChrA09g005073*	synonymous SNV
42421289	T	C	*ChrA09g005073*	synonymous SNV
42421433	T	C	*ChrA09g005073*	synonymous SNV
42421451	G	A	*ChrA09g005073*	synonymous SNV
42421604	T	C	*ChrA09g005073*	synonymous SNV
42421648	G	A	*ChrA09g005073*	intronic
42421652	G	T	*ChrA09g005073*	intronic
42423444	C	G	*ChrA09g005074*	nonsynonymous SNV
42423481	T	C	*ChrA09g005074*	nonsynonymous SNV
42423974	C	T	*ChrA09g005074*	synonymous SNV
42424788	T	A	*ChrA09g005074*	downstream
42424974	G	GT	*ChrA09g005074*	downstream
42427478	A	A,G	*ChrA09g005074-ChrA09g005075*	intergenic
42427497	T	T,TC	*ChrA09g005074-ChrA09g005075*	intergenic
42427513	G	T	*ChrA09g005074-ChrA09g005075*	intergenic
42427516	AG	AG,A	*ChrA09g005074-ChrA09g005075*	intergenic
42427519	G	G,A	*ChrA09g005074-ChrA09g005075*	intergenic
42427550	T	T,G	*ChrA09g005074-ChrA09g005075*	intergenic
42427564	A	A,G	*ChrA09g005074-ChrA09g005075*	intergenic
42427610	T	T,C	*ChrA09g005074-ChrA09g005075*	intergenic
42427746	G	G,A	*ChrA09g005074-ChrA09g005075*	intergenic
42427908	G	A	*ChrA09g005074-ChrA09g005075*	intergenic
42427920	C	T	*ChrA09g005074-ChrA09g005075*	intergenic
42428103	C	T	*ChrA09g005074-ChrA09g005075*	intergenic
42428201	C	T	*ChrA09g005074-ChrA09g005075*	intergenic
42428665	G	A	*ChrA09g005074-ChrA09g005075*	intergenic
42428963	TG	T	*ChrA09g005074-ChrA09g005075*	intergenic
42428978	AT	A	*ChrA09g005074-ChrA09g005075*	intergenic
42428984	T	G	*ChrA09g005074-ChrA09g005075*	intergenic
42429350	T	T,C	*ChrA09g005074-ChrA09g005075*	intergenic
42429367	T	T,TAA	*ChrA09g005074-ChrA09g005075*	intergenic
42429395	G	G,A	*ChrA09g005074-ChrA09g005075*	intergenic
42429422	A	G	*ChrA09g005074-ChrA09g005075*	intergenic

42430669	G	GA	*ChrA09g005074-ChrA09g005075*	intergenic
42441033	A	G	*ChrA09g005074-ChrA09g005075*	intergenic
42441101	A	T	*ChrA09g005074-ChrA09g005075*	intergenic
42441539	A	AAT	*ChrA09g005074-ChrA09g005075*	intergenic
42442050	G	GTATA	*ChrA09g005074-ChrA09g005075*	intergenic
42442715	T	A	*ChrA09g005074-ChrA09g005075*	intergenic
42443395	A	C	*ChrA09g005074-ChrA09g005075*	intergenic
42443474	T	T,TA	*ChrA09g005074-ChrA09g005075*	intergenic
42443505	G	A	*ChrA09g005074-ChrA09g005075*	intergenic
42443519	AT	A	*ChrA09g005074-ChrA09g005075*	intergenic
42443528	T	C	*ChrA09g005074-ChrA09g005075*	intergenic
42443570	T	C	*ChrA09g005074-ChrA09g005075*	intergenic
42443571	C	T	*ChrA09g005074-ChrA09g005075*	intergenic
42443817	T	TGACAA	*ChrA09g005075*	upstream
42443819	T	TAAAA	*ChrA09g005075*	upstream
42443820	C	CGAAACGGAATAAGTATAAAACTATTTTAAAAATACTGA	*ChrA09g005075*	upstream
42444392	G	C	*ChrA09g005075*	upstream
42444555	TA	T	*ChrA09g005075*	upstream
42444893	T	C	*ChrA09g005075*	upstream
42444899	T	G	*ChrA09g005075*	upstream
42444900	C	T	*ChrA09g005075*	upstream
42444925	C	T	*ChrA09g005075*	upstream
42444965	G	A	*ChrA09g005075*	upstream
42445038	A	G	*ChrA09g005075*	upstream
42445773	C	A	*ChrA09g005075*	upstream
42445797	C	T	*ChrA09g005075*	upstream
42446400	A	T	*ChrA09g005075*	UTR5
42446496	T	C	*ChrA09g005075*	intronic
42446587	A	G	*ChrA09g005075*	intronic
42446600	G	T	*ChrA09g005075*	intronic
42446623	C	G	*ChrA09g005075*	nonsynonymous SNV
42446630	A	G	*ChrA09g005075*	synonymous SNV
42446914	T	C	*ChrA09g005075*	synonymous SNV
42447087	G	C	*ChrA09g005075*	intronic
42447089	G	A	*ChrA09g005075*	intronic
42447096	A	AT	*ChrA09g005075*	intronic
42447104	GT	G	*ChrA09g005075*	intronic
42447138	T	A	*ChrA09g005075*	synonymous SNV
42447189	C	T	*ChrA09g005075*	synonymous SNV
42447207	C	A	*ChrA09g005075*	synonymous SNV

42447368	A	T	*ChrA09g005075*	intronic
42447430	T	TG	*ChrA09g005075*	intronic
42447482	A	C	*ChrA09g005075*	synonymous SNV
42447740	T	A	*ChrA09g005075*	synonymous SNV
42447782	A	C	*ChrA09g005075*	intronic
42447832	C	T	*ChrA09g005075*	intronic
42447834	G	T	*ChrA09g005075*	intronic
42447866	A	G	*ChrA09g005075*	nonsynonymous SNV
42447930	G	A	*ChrA09g005075*	nonsynonymous SNV
42448310	C	T	*ChrA09g005075*	synonymous SNV
42448311	A	G	*ChrA09g005075*	nonsynonymous SNV
42448367	G	GCCTCCT	*ChrA09g005075*	nonframeshift insertion
42449739	A	T	*ChrA09g005075*	downstream
42449803	G	A	*ChrA09g005075*	downstream
42449834	A	T	*ChrA09g005075*	downstream
42449837	T	C	*ChrA09g005075*	downstream
42449875	C	G	*ChrA09g005075*	downstream
42449945	A	T	*ChrA09g005075*	downstream
42449963	G	C	*ChrA09g005075*	downstream
42450127	GGAA	G	*ChrA09g005075*	downstream
42450254	A	G	*ChrA09g005075*	downstream
42450531	T	A	*ChrA09g005075*	downstream
42451483	T	G	*ChrA09g005076*	upstream
42452237	AG	A	*ChrA09g005076*	upstream
42452288	A	AAGT	*ChrA09g005076*	upstream
42452687	CA	C	*ChrA09g005076*	UTR5
42452695	T	G	*ChrA09g005076*	UTR5
42452751	ACT	A	*ChrA09g005076*	UTR5
42453055	A	C	*ChrA09g005076*	UTR5
42453149	A	G	*ChrA09g005076*	UTR5
42453181	T	TCAGGTCTTA	*ChrA09g005076*	UTR5
42454404	TTATTGG	T	*ChrA09g005076*	UTR3
42454412	ATCTCT	A	*ChrA09g005076*	UTR3
42454559	G	GTT	*ChrA09g005076*	UTR3
42455025	C	T	*ChrA09g005076*	downstream
42455074	TTTTG	T	*ChrA09g005076*	downstream
42455173	A	T	*ChrA09g005076*	downstream
42455208	C	A	*ChrA09g005076*	downstream
42455424	G	A	*ChrA09g005076*	downstream
42455748	A	G	*ChrA09g005076*	downstream

42455766	GCGCT	G	*ChrA09g005076*	downstream
42455777	CTT	C	*ChrA09g005076*	downstream
42455827	C	CT	*ChrA09g005076*	downstream
42455871	C	T	*ChrA09g005076*	downstream
42455874	T	C	*ChrA09g005076*	downstream
42456294	C	G	*ChrA09g005076*	downstream
42457186	C	A	*ChrA09g005076-ChrA09g005077*	intergenic
42457430	G	C	*ChrA09g005076-ChrA09g005077*	intergenic
42457462	C	T	*ChrA09g005076-ChrA09g005077*	intergenic
42457472	A	G	*ChrA09g005076-ChrA09g005077*	intergenic
42457495	T	A	*ChrA09g005076-ChrA09g005077*	intergenic
42457497	T	A	*ChrA09g005076-ChrA09g005077*	intergenic
42457576	T	A	*ChrA09g005076-ChrA09g005077*	intergenic
42457705	G	T	*ChrA09g005077*	downstream
42457721	A	T	*ChrA09g005077*	downstream
42457736	A	G	*ChrA09g005077*	downstream
42457756	C	T	*ChrA09g005077*	downstream
42457767	G	A	*ChrA09g005077*	downstream
42457868	C	T	*ChrA09g005077*	downstream
42457869	G	A	*ChrA09g005077*	downstream
42457918	A	G	*ChrA09g005077*	downstream
42458036	A	G	*ChrA09g005077*	downstream
42458142	G	T	*ChrA09g005077*	downstream
42458149	A	G	*ChrA09g005077*	downstream
42458240	T	C	*ChrA09g005077*	downstream
42458259	A	T	*ChrA09g005077*	downstream
42458274	C	T	*ChrA09g005077*	downstream
42458276	C	CGAATA	*ChrA09g005077*	downstream
42458424	G	A	*ChrA09g005077*	downstream
42458557	A	AGCC	*ChrA09g005077*	downstream
42459025	A	AT	*ChrA09g005077*	downstream
42459390	A	G	*ChrA09g005077*	downstream
42459534	T	T,A	*ChrA09g005077*	downstream
42459584	C	A	*ChrA09g005077*	downstream
42459628	A	G	*ChrA09g005077*	downstream
42461422	T	T,C	*ChrA09g005077*	intronic
42461428	CT	CT,C	*ChrA09g005077*	intronic
42461437	T	T,C	*ChrA09g005077*	intronic
42461453	A	A,AT	*ChrA09g005077*	intronic
42462051	T	C	*ChrA09g005077*	synonymous SNV

42462109	G	T	*ChrA09g005077*	nonsynonymous SNV
42462123	A	T	*ChrA09g005077*	synonymous SNV
42462381	C	T	*ChrA09g005077*	synonymous SNV
42462438	A	G	*ChrA09g005077*	synonymous SNV
42462462	A	T	*ChrA09g005077*	synonymous SNV
42462465	G	C	*ChrA09g005077*	synonymous SNV
42462474	C	T	*ChrA09g005077*	synonymous SNV
42462624	A	C	*ChrA09g005077*	synonymous SNV
42462636	C	G	*ChrA09g005077*	synonymous SNV
42462693	C	G	*ChrA09g005077*	synonymous SNV
42462705	T	C	*ChrA09g005077*	synonymous SNV
42462759	G	C	*ChrA09g005077*	nonsynonymous SNV
42462987	A	T	*ChrA09g005077*	synonymous SNV
42463029	G	C	*ChrA09g005077*	synonymous SNV
42478555	G	A	*ChrA09g005080-ChrA09g005081*	intergenic
42497405	A	G	*ChrA09g005080-ChrA09g005081*	intergenic
42498448	G	A	*ChrA09g005080-ChrA09g005081*	intergenic
42503880	CA	C	*ChrA09g005080-ChrA09g005081*	intergenic
42518959	T	C	*ChrA09g005080-ChrA09g005081*	intergenic
42532848	TG	T	*ChrA09g005086*	frameshift deletion
42538885	C	CA	*ChrA09g005087*	downstream
42539416	G	A	*ChrA09g005087*	downstream
42559787	G	A	*ChrA09g005091*	nonsynonymous SNV
42560038	A	AACT	*ChrA09g005091*	UTR5
42560077	CTTTT	C	*ChrA09g005091*	UTR5
42560116	A	G	*ChrA09g005091*	UTR5
42560143	A	ATT	*ChrA09g005091*	UTR5
42560167	G	T	*ChrA09g005091*	UTR5
42560172	G	T	*ChrA09g005091*	UTR5
42560613	A	G	*ChrA09g005091*	upstream
42563290	G	C	*ChrA09g005091-ChrA09g005092*	intergenic
42563311	C	T	*ChrA09g005091-ChrA09g005092*	intergenic
42563852	C	A	*ChrA09g005091-ChrA09g005092*	intergenic
42564400	A	G	*ChrA09g005091-ChrA09g005092*	intergenic
42564409	TA	T	*ChrA09g005091-ChrA09g005092*	intergenic
42564663	C	CAATGCTT	*ChrA09g005091-ChrA09g005092*	intergenic
42565101	A	T	*ChrA09g005091-ChrA09g005092*	intergenic
42565733	G	T	*ChrA09g005091-ChrA09g005092*	intergenic
42565857	A	AAG	*ChrA09g005091-ChrA09g005092*	intergenic
42565863	TTA	T	*ChrA09g005091-ChrA09g005092*	intergenic

42565887	T	G	*ChrA09g005091-ChrA09g005092*	intergenic
42565896	T	TCTGTAAGACGTAGTAAATC	*ChrA09g005092*	upstream
42566006	T	TATC	*ChrA09g005092*	upstream
42566068	C	A	*ChrA09g005092*	upstream
42566156	GTAATACGTAGACCTTATCTAT	G	*ChrA09g005092*	upstream
42566194	A	AC	*ChrA09g005092*	upstream
42566729	A	G	*ChrA09g005092*	upstream
42566810	T	C	*ChrA09g005092*	upstream
42566871	GTT	G	*ChrA09g005092*	upstream
42566935	G	T	*ChrA09g005092*	upstream
42567754	G	GA	*ChrA09g005092*	upstream
42567916	A	T	*ChrA09g005092*	UTR5
42568848	G	C	*ChrA09g005092*	nonsynonymous SNV
42571869	C	CTTTTGAAGGAAACAATCATACAAGA	*ChrA09g005093*	UTR3
42571934	C	T	*ChrA09g005093*	UTR3
42571969	TGAAC,T	T	*ChrA09g005093*	UTR3
42572414	ATGC	A	*ChrA09g005093*	nonframeshift deletion
42572463	C	A	*ChrA09g005093*	UTR5
42572466	T	C	*ChrA09g005093*	UTR5
42576566	C	A	*ChrA09g005094-ChrA09g005095*	intergenic
42576618	T	C	*ChrA09g005094-ChrA09g005095*	intergenic
42577894	T	C	*ChrA09g005094-ChrA09g005095*	intergenic
42578237	C	T	*ChrA09g005094-ChrA09g005095*	intergenic
42578253	C	T	*ChrA09g005094-ChrA09g005095*	intergenic
42578331	A	C	*ChrA09g005094-ChrA09g005095*	intergenic
42578334	G	A	*ChrA09g005094-ChrA09g005095*	intergenic
42578385	C	T	*ChrA09g005094-ChrA09g005095*	intergenic
42578392	T	C	*ChrA09g005094-ChrA09g005095*	intergenic
42578429	G	T	*ChrA09g005094-ChrA09g005095*	intergenic
42578442	T	C	*ChrA09g005094-ChrA09g005095*	intergenic
42582990	G	T	*ChrA09g005095*	upstream
42583527	T	C	*ChrA09g005095*	upstream
42584239	TTG	T	*ChrA09g005095*	intronic
42584242	C	CT	*ChrA09g005095*	intronic
42584245	AT	A	*ChrA09g005095*	intronic
42584412	C	T	*ChrA09g005095*	intronic
42585567	A	G	*ChrA09g005095*	intronic
42585665	T	C	*ChrA09g005095*	synonymous SNV
42586671	G	A	*ChrA09g005095*	synonymous SNV
42586813	C	A	*ChrA09g005095*	intronic

42586828	G	A	*ChrA09g005095*	intronic
42586838	A	A,AATATCTGTGGTG	*ChrA09g005095*	intronic
42586847	C	C,T	*ChrA09g005095*	intronic
42587169	C	T	*ChrA09g005095*	synonymous SNV
42587731	A	T	*ChrA09g005095*	synonymous SNV
42587809	A	T	*ChrA09g005095*	synonymous SNV
42587812	C	T	*ChrA09g005095*	synonymous SNV
42587824	G	A	*ChrA09g005095*	synonymous SNV
42587827	A	G	*ChrA09g005095*	synonymous SNV
42587953	T	A	*ChrA09g005095*	intronic
42587960	A	A,C	*ChrA09g005095*	intronic
42587961	T	T,TG	*ChrA09g005095*	intronic
42587967	C	C,T	*ChrA09g005095*	intronic
42588201	T	T,TGCAAAGAAACA	*ChrA09g005095*	intronic
42588202	T	T,TG	*ChrA09g005095*	intronic
42588204	T	T,C	*ChrA09g005095*	intronic
42588218	A	A,G	*ChrA09g005095*	intronic
42588242	C	C,T	*ChrA09g005095*	intronic
42588248	T	A	*ChrA09g005095*	intronic
42588255	A	T	*ChrA09g005095*	intronic
42588256	C	T	*ChrA09g005095*	intronic
42588259	G	GT	*ChrA09g005095*	intronic
42588260	C	A	*ChrA09g005095*	intronic
42588266	T	A	*ChrA09g005095*	intronic
42588316	T	C	*ChrA09g005095*	synonymous SNV
42588340	C	A	*ChrA09g005095*	synonymous SNV
42588352	C	T	*ChrA09g005095*	synonymous SNV
42588394	T	A	*ChrA09g005095*	synonymous SNV
42588404	T	C	*ChrA09g005095*	synonymous SNV
42588436	A	G	*ChrA09g005095*	synonymous SNV
42588448	T	C	*ChrA09g005095*	synonymous SNV
42588478	C	A	*ChrA09g005095*	synonymous SNV
42588524	G	C	*ChrA09g005095*	intronic
42588532	T	TAA	*ChrA09g005095*	intronic
42588539	A	ATAT	*ChrA09g005095*	intronic
42588540	AC	A	*ChrA09g005095*	intronic
42588544	G	C	*ChrA09g005095*	intronic
42588570	T	C	*ChrA09g005095*	intronic
42588573	T	G	*ChrA09g005095*	intronic
42588608	T	A	*ChrA09g005095*	synonymous SNV

42588758	G	A	*ChrA09g005095*	synonymous SNV
42588761	A	G	*ChrA09g005095*	synonymous SNV
42588770	T	G	*ChrA09g005095*	synonymous SNV
42588773	G	T	*ChrA09g005095*	synonymous SNV
42588833	T	A	*ChrA09g005095*	synonymous SNV
42588846	G	T	*ChrA09g005095*	nonsynonymous SNV
42588897	A	AT	*ChrA09g005095*	intronic
42588931	T	G	*ChrA09g005095*	intronic
42588934	C	T	*ChrA09g005095*	intronic
42589458	T	A	*ChrA09g005095*	intronic
42589514	T	A	*ChrA09g005095*	intronic
42589515	T	C	*ChrA09g005095*	intronic
42589533	G	A	*ChrA09g005095*	intronic
42590007	C	T	*ChrA09g005096*	synonymous SNV
42590137	T	G	*ChrA09g005096*	nonsynonymous SNV
42597619	T	C	*ChrA09g005100*	nonsynonymous SNV
42599112	C	CA	*ChrA09g005100*	intronic
42600825	G	A	*ChrA09g005101*	UTR5
42600843	C	A	*ChrA09g005101*	UTR5
42600944	C	A	*ChrA09g005101*	synonymous SNV
42600955	T	T,C	*ChrA09g005101*	nonsynonymous SNV
42601269	T	T,G	*ChrA09g005101*	synonymous SNV
42602980	T	C	*ChrA09g005101*	intronic
42603129	T	A	*ChrA09g005101*	UTR3
42604171	T	A	*ChrA09g005102*	UTR3
42604179	C	T	*ChrA09g005102*	UTR3
42604455	C	A	*ChrA09g005102*	synonymous SNV
42605120	T	C	*ChrA09g005102*	UTR5
42609033	C	T	*ChrA09g005104*	synonymous SNV
42613008	C	CT	*ChrA09g005106*	UTR5
42613034	C	T	*ChrA09g005106*	UTR5
42613127	A	G	*ChrA09g005106*	nonsynonymous SNV
42613129	C	G	*ChrA09g005106*	nonsynonymous SNV
42613236	T	A	*ChrA09g005106*	nonsynonymous SNV
42613281	T	TTGGAACA	*ChrA09g005106*	intronic
42613300	A	T	*ChrA09g005106*	intronic
42613301	A	T	*ChrA09g005106*	intronic
42613435	T	G	*ChrA09g005106*	intronic
42613627	T,C	C	*ChrA09g005106*	nonsynonymous SNV
42613639	A,T	T	*ChrA09g005106*	intronic

42614777	A	G	*ChrA09g005106*	intronic
42614789	G	C	*ChrA09g005106*	intronic
42614791	G	A	*ChrA09g005106*	intronic
42615762	C	T	*ChrA09g005107*	synonymous SNV
42615821	C	C,G	*ChrA09g005107*	nonsynonymous SNV
42615845	C	T	*ChrA09g005107*	nonsynonymous SNV
42615901	C	T	*ChrA09g005107*	nonsynonymous SNV
42615902	C	T	*ChrA09g005107*	nonsynonymous SNV
42615933	C	G	*ChrA09g005107*	synonymous SNV
42615970	A	G	*ChrA09g005107*	nonsynonymous SNV
42616013	C	T	*ChrA09g005107*	nonsynonymous SNV
42616035	G	G,T	*ChrA09g005107*	nonsynonymous SNV
42616083	G	C	*ChrA09g005106-ChrA09g005107*	intronic
42616183	G	G,A	*ChrA09g005107*	synonymous SNV
42616274	G	A	*ChrA09g005106-ChrA09g005107*	intronic
42620503	T	TTCTC	*ChrA09g005108*	upstream
42621051	ATGAGGCGACCAAG	A	*ChrA09g005108*	upstream
42621257	A	C	*ChrA09g005108*	upstream
42624120	TA	T	*ChrA09g005109*	UTR3
42624672	G	A	*ChrA09g005109*	synonymous SNV
42625329	A	T	*ChrA09g005109*	intronic
42626341	A	A,C	*ChrA09g005109*	intronic
42626344	G	G,A	*ChrA09g005109*	intronic
42626346	C	C,CTCA	*ChrA09g005109*	intronic
42626354	G	A	*ChrA09g005109*	intronic
42626362	C	T	*ChrA09g005109*	intronic
42626412	A	G	*ChrA09g005109*	intronic
42628084	A	G	*ChrA09g005110*	UTR3
42628099	A	G	*ChrA09g005110*	UTR3
42628103	T	G	*ChrA09g005110*	UTR3
42628137	A	G	*ChrA09g005110*	UTR3
42628217	G	C	*ChrA09g005110*	UTR3
42628361	A	C	*ChrA09g005110*	intronic
42628392	C	G	*ChrA09g005110*	intronic
42628395	T	C	*ChrA09g005110*	intronic
42628406	AT	A	*ChrA09g005110*	intronic
42628424	A	T	*ChrA09g005110*	intronic
42628602	G,A	A	*ChrA09g005110*	intronic

Table S3. Annotation of the predicted genes with exonic (splicing) variants related to *qSD.A9-1* (*qSL.A9*).

Gene ID	Start base - end base (bp)[a]	Chain	Predicted function[b]
chrA09g005038	42228775-42230395	Forward	Bestrophin, RFP-TM, chloride channel
chrA09g005039	42230607-42232064	Reverse	Pentatricopeptide (PPR) repeat
chrA09g005040	42232704-42233305	Forward	Protein of unknown function
chrA09g005041	42233780-42236057	Reverse	Domain of unknown function (DUF4378)
chrA09g005043	42251412-42253616	Reverse	Skp1 family, dimerisation domain
chrA09g005044	42249423-42250932	Forward	–
chrA09g005046	42266768-42271038	Forward	Pyridoxal-phosphate dependent enzyme
chrA09g005047	42273808-42275283	Forward	SNARE domain
chrA09g005048	42286043-42286564	Reverse	Ring finger domain
chrA09g005049	42291798-42294644	Reverse	2OG-Fe(II) oxygenase superfamily
chrA09g005050	42295840-42303051	Forward	Ribosome control protein 1
chrA09g005051	42304668-42306966	Forward	Glycosyl hydrolases family 28
chrA09g005052	42317619-42319582	Forward	Aminotransferase class I and II
chrA09g005053	42320445-42321636	Forward	–
chrA09g005054	42322647-42324762	Reverse	Aminotransferase class I and II
chrA09g005055	42331464-42332847	Reverse	Ketopantoate hydroxymethyltransferase
chrA09g005056	42334819-42335478	Forward	Ring finger domain
chrA09g005057	42339244-42343199	Reverse	–
chrA09g005058	42344565-42345908	Forward	Fatty acid desaturase
chrA09g005059	42351674-42352891	Forward	F-box-like
chrA09g005060	42354956-42355822	Forward	Plastocyanin-like domain
chrA09g005061	42359156-42361732	Forward	BTB/Kelch-associated
chrA09g005063	42364930-42366436	Forward	Aldose 1-epimerase
chrA09g005068	42385655-42386086	Forward	–
chrA09g005069	42389673-42392397	Forward	Probable zinc-ribbon domain
chrA09g005070	42398590-42403559	Forward	–
chrA09g005071	42413482-42416154	Reverse	Plant phosphoribosyltransferase C-terminal
chrA09g005072	42416897-42418237	Reverse	Ring finger domain
chrA09g005073	42419533-42421930	Reverse	Cytochrome b561/ferric reductase transmembrane
chrA09g005074	42423381-42424249	Forward	Divergent PAP2 family
chrA09g005075	42446438-42449059	Forward	Auxin response factor
chrA09g005077	42459639-42463361	Reverse	RNA recognition motif (RRM) domain
chrA09g005086	42532079-42533390	Reverse	No apical meristem (NAM) protein
chrA09g005091	42551291-42559952	Reverse	Protein kinase domain
chrA09g005092	42568247-42569125	Forward	B3 DNA binding domain
chrA09g005093	42572083-42572439	Reverse	–

chrA09g005095	42584522-42589684	Forward	Inositol hexakisphosphate
chrA09g005096	42589959-42590734	Reverse	Cupin
chrA09g005100	42597552-42599204	Reverse	Haloacid dehalogenase-like hydrolase
chrA09g005101	42600903-42603126	Forward	Pectinacetylesterase
chrA09g005102	42604338-42605075	Reverse	–
chrA09g005104	42608908-42609860	Forward	Protein phosphatase 2A regulatory B subunit (B56 family)
chrA09g005106	42613067-42615266	Forward	Snf7
chrA09g005107	42615681-42616997	Reverse	–
chrA09g005109	42624276-42626650	Reverse	Glycosyl hydrolases family 28

[a]Physical position of predicted gene obtained from the reference genome of Ningyou7. [b]Predicted function of candidate gene based on Ningyou7 annotation information. "–" indicates no putative conserved domains have been detected.
